# Inferring Genome-Wide Correlations of Mutation Fitness Effects between Populations

**DOI:** 10.1093/molbev/msab162

**Published:** 2021-05-27

**Authors:** Xin Huang, Alyssa Lyn Fortier, Alec J Coffman, Travis J Struck, Megan N Irby, Jennifer E James, José E León-Burguete, Aaron P Ragsdale, Ryan N Gutenkunst

**Affiliations:** 1 Department of Molecular and Cellular Biology, University of Arizona, Tucson, AZ, USA; 2 Department of Biology, Stanford University, Stanford, CA, USA; 3 Department of Chemistry, University of Pennsylvania, Philadelphia, PA, USA; 4 Center for Genomic Sciences, National Autonomous University of Mexico, MR, Mexico; 5 Department of Human Genetics, McGill University, Montreal, QC, Canada

**Keywords:** population genetics, distribution of fitness effects, population divergence

## Abstract

The effect of a mutation on fitness may differ between populations depending on environmental and genetic context, but little is known about the factors that underlie such differences. To quantify genome-wide correlations in mutation fitness effects, we developed a novel concept called a joint distribution of fitness effects (DFE) between populations. We then proposed a new statistic *w* to measure the DFE correlation between populations. Using simulation, we showed that inferring the DFE correlation from the joint allele frequency spectrum is statistically precise and robust. Using population genomic data, we inferred DFE correlations of populations in humans, *Drosophila melanogaster*, and wild tomatoes. In these species, we found that the overall correlation of the joint DFE was inversely related to genetic differentiation. In humans and *D. melanogaster*, deleterious mutations had a lower DFE correlation than tolerated mutations, indicating a complex joint DFE. Altogether, the DFE correlation can be reliably inferred, and it offers extensive insight into the genetics of population divergence.

## Introduction

New mutations that alter fitness are the key input into the evolutionary process. Typically, the majority of new mutations are deleterious or nearly neutral, and only a small minority are adaptive. These three categories constitute a continuum of fitness effects—the distribution of fitness effects (DFE) of new mutations (Eyre-Walker and Keightley 2007). The DFE is central to many theoretical evolutionary topics, such as the maintenance of genetic variation (Charlesworth 1994) and the evolution of recombination (Barton 1995), in addition to being key to applied evolutionary topics, such as the emergence of pathogens (Gandon et al. 2013) and the genetic architecture of complex disease (Durvasula and Lohmueller 2021).

The DFE can be quantified by either experimental approaches or statistical inference. Experimental approaches measure the DFE using random mutagenesis (Elena et al. 1998) or mutation accumulation (Fry et al. 1999); however, these approaches are limited to studying a small number of mutations. Most of our knowledge regarding the DFE has come from statistical inferences based on contemporary patterns of natural genetic variation. In these inferences, genetic data are typically summarized by the allele frequency spectrum (AFS; also known as the site frequency spectrum, SFS). In some methods, a demographic model is inferred from the AFS of putatively neutral variants, and the DFE is estimated from the AFS of variants under selection, conditional on the best fit demographic model (Eyre-Walker et al. 2006; Keightley and Eyre-Walker 2007; Boyko et al. 2008; Kim et al. 2017). In other methods, the background pattern of variation is accounted for by the inclusion of nuisance parameters when fitting a DFE model to the AFS of variants under selection (Eyre-Walker et al. 2006; Tataru et al. 2017; Barton and Zeng 2018). In an alternative approach, a recent study applied approximate Bayesian computation to simultaneously infer the DFE and a demographic model (Johri et al. 2020). Moreover, a linear regression method can be used to infer the DFE from nucleotide diversity (James et al. 2017). These approaches has been applied to numerous organisms, including plants (Chen et al. 2017; Huber et al. 2018; Chen et al. 2020), *Drosophila melanogaster* (Keightley and Eyre-Walker 2007; Castellano et al. 2017; Huber et al. 2017; Barton and Zeng 2018; Johri et al. 2020), and primates (Boyko et al. 2008; Huber et al. 2017; Kim et al. 2017; Ma et al. 2013; Castellano et al. 2019).

Using these inference methods, several studies have found evidence for differences in DFEs among different populations (Boyko et al. 2008; Ma et al. 2013; Kim et al. 2017; Castellano et al. 2019; Tataru and Bataillon 2019). These studies, however, have been limited by the implicit assumption that the fitness effects of a given mutation in different populations are independent draws from distinct DFEs. Moreover, these studies only compared DFEs from the AFS of single populations and therefore cannot investigate differences in fitness effects in new environments after population divergence. Intuitively, we expect the fitness effects of a given mutation in different contexts to be correlated. Wang et al. (2009) experimentally measured the fitness effects of twenty dominant mutations in two environments in *D. melanogaster* and found a strong positive correlation. But the generality of their results is unclear, and it is not known what factors affect the strength of the correlation.

Considering deleterious mutations, here we developed a novel concept called the joint DFE of new mutations, which can be inferred from the joint AFS of pairs of populations. We then defined the correlation of mutation fitness effects between populations using the joint DFE. With simulation, we showed that inferring the joint DFE and correlation requires only modest sample sizes and is robust to many forms of model misspecification. We then applied our approach to data from humans, *D. melanogaster*, and wild tomatoes. We found that the correlation of mutation fitness effects between populations is lowest in wild tomatoes and highest in humans. In *D. melanogaster* and wild tomatoes, we found differences in the correlation among genes with different functions. We also found that mutations with more deleterious effects exhibit lower correlations. Together, our results show that the joint DFE and correlation of mutation fitness effects offer new insight into the population genetics of these species.

## Results

### Definition

**Fig. 1.**
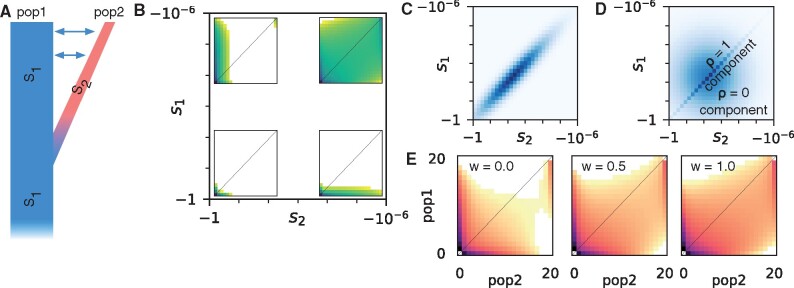
The joint allele frequency spectrum (AFS) and joint distribution of fitness effects (DFE). (*A*) We considered populations that have recently diverged with gene flow between them. Some genetic variants will have a different effect on fitness in the diverged population (*s*_2_) than in the ancestral population (*s*_1_). (*B*) The joint DFE is defined over pairs of selection coefficients (*s*_1_, *s*_2_). Insets show the joint AFS for pairs of variants that are strongly or weakly deleterious in each population. In each spectrum, the number of segregating variants at a given pair of allele frequencies is exponential with the color depth. (*C*) One potential model for the joint DFE is a bivariate lognormal distribution, illustrated here for strong correlation. (*D*) We focus on a model in which the joint DFE is a mixture of components corresponding to equality (*ρ* = 1) and independence (*ρ* = 0) of fitness effects. (*E*) As illustrated by these simulated allele frequency spectra, stronger correlations of mutation fitness effects lead to more shared polymorphism. Here, *w* is the weight of the *ρ* = 1 component in the mixture model.

To define the joint DFE, we considered two populations that have recently diverged, one of which may have entered a new environment (fig. 1*A*). We also considered that a mutation has selection coefficient *s*_1_ in the ancestral population and *s*_2_ in the recently diverged population. For two populations, the joint AFS is a matrix in which each entry *i*, *j* corresponds to the number of variants observed at frequency *i* in population 1 and *j* in population 2 in a sequenced sample of individuals from the two populations. Different combinations of *s*_1_ and *s*_2_ lead to distinct patterns in the joint AFS (fig. 1*B*). We refer to the joint probability distribution for (*s*_1_, *s*_2_) as the joint DFE (fig. 1*C*), and we refer to the marginal probability distributions for *s*_1_ or *s*_2_ as the marginal DFEs for population 1 or population 2, respectively. The observed AFS from a pair of populations results from integrating spectra for different values of *s*_1_ and *s*_2_ over the joint DFE.

Little is known about the shape of the joint DFE, so we considered multiple parametric models. The best fit DFEs for single populations tend to be lognormal or gamma distributions (Boyko et al. 2008), although discrete distributions may sometimes fit better (Kousathanas and Keightley 2013; Johri et al. 2020). We first considered a bivariate lognormal distribution (fig. 1*C*), because it has an easily interpretable correlation coefficient. However, accurate numerical integration over the bivariate lognormal distribution becomes challenging when the correlation coefficient approaches one, because probability density becomes concentrated in a small number of sampled grid points (supplementary fig. S1, Supplementary Material online). We also considered another popular probability distribution for modeling DFEs, the gamma distribution, but there are multiple ways of defining a bivariate gamma distribution (Nadarajah and Gupta 2006). We thus focused on a mixture model that consisted of a component corresponding to perfect correlation with weight *w*, and a component corresponding to zero correlation with weight (1−w) (fig. 1*D*). To limit the complexity of the model, we assumed that the marginal DFEs were identical for both populations. In this case, the correlation of the overall distribution is equal to the mixture proportion *w*. We thus interpret and discuss *w* as a DFE correlation coefficient.

The DFE correlation profoundly affects the expected AFS (fig. 1*E*). Qualitatively, if the correlation is low, there is little shared high-frequency polymorphism. In this case, alleles that are nearly neutral in one population are often deleterious in the other, driving their frequencies lower in that population. If the correlation of the joint DFE is larger, more shared polymorphism is preserved. To calculate the expected AFS for a given demographic model and DFE, we first cached calculations of the expected AFS for a grid of selection coefficient pairs. Assuming independence among sites, the expectation for the full DFE is then an integration over values of *s*_1_, *s*_2_, weighted by the DFE (supplementary fig. S1, Supplementary Material online) (Ragsdale et al. 2016; Kim et al. 2017). We based our approach on the fitdadi framework developed by Kim et al. (2017), and our approach is integrated into our dadi software (Gutenkunst et al. 2009). More detail can be found in the Materials and Methods section.

### Simulation

We focused our simulation studies on cases in which the correlation of the DFE was high, because those cases turned out to be most relevant to our empirical analyses.

To evaluate the precision of our approach, we first stochastically simulated unlinked single nucleotide polymorphisms (SNPs) under a known demographic model (supplementary table S1 and fig. S2, Supplementary Material online) and a symmetric lognormal mixture model for the joint DFE (fig. 1; eqs. 4 and 6). We then inferred the three joint DFE parameters: the mean ***μ*** and standard deviation ***σ*** of the marginal lognormal distributions and the DFE correlation *w*. The demographic and joint DFE parameters for these simulations were similar to those we later inferred for human populations under a demographic model of divergence, growth, and migration. When we fit the joint DFE to these simulated data, we found that the variance of the inferred parameters grew only slowly as the sample size decreased (supplementary fig. S3*A*, Supplementary Material online). This suggests that only modest sample sizes are necessary to confidently infer the joint DFE, similar to how only modest sample sizes are necessary to infer the mean and variance of the univariate DFE (Keightley and Eyre-Walker 2010).

Because our inference approach focuses on shared variation, we expected precision to depend on the divergence time between the populations. To test this, we simulated data sets with sample size similar to our real Drosophila data and varied the divergence time in the demographic model. We found that the variance of the inferred ***μ*** and ***σ*** parameters was always small (supplementary fig. S3*B* and *C*, Supplementary Material online), but the variance of the inferred DFE correlation *w* depended on the divergence time (supplementary fig. S3*B* and *C*, Supplementary Material online). That variance in *w* was large for small divergence times ($T=10^{-4}$). This is expected, because in this case selection has had little time act differently in the two populations. That variance in *w* was also large if the divergence time was large and there was no migration between the populations (supplementary fig. S3*C*, Supplementary Material online). This is also expected, because in this scenario there is little shared variation between populations. However, the variance of the inferred DFE correlation *w* was small when the divergence time was between $10^{-3}$ and 10_**°**_ (supplementary fig. S3*B* and *C*, Supplementary Material online). Moreover, the variance of *w* was not large unless *F_ST_* in the simulated data was substantially larger than found in the empirical data we analyzed. Ancestral state misidentification could bias our inference (Baudry and Depaulis 2003). To account for this, in our empirical analyses we included a model parameter for such misidentification. Tests with simulated data showed that the degree of misidentification could be precisely inferred (supplementary fig. S4, Supplementary Material online), and including this parameter in our model does not strongly affect other inferences (supplementary table S14, Supplementary Material online).

Having found good precision for our inference, we then turned to testing the robustness of our inference to model misspecification. Since these tests focused on biases in the average inference, we did not stochastically sample data for these analyses, but rather used the expected AFS under each scenario as the data.

**Fig. 2.**
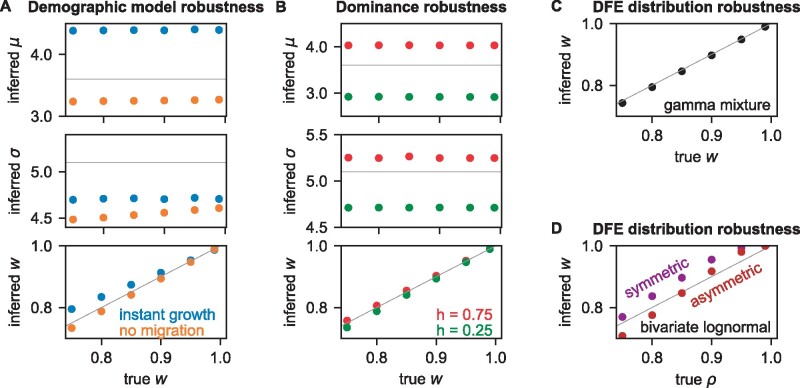
Robustness of joint DFE inference to model misspecification. Simulated neutral and selected data were generated under a demographic model with exponential growth and migration (supplementary table S1, Supplementary Material online), and lognormal mixture DFE models were fit to the data. The DFE parameters are: *μ*, the mean log population-scaled selection coefficient; *σ*, the standard deviation of those log coefficients; and *w*, the correlation of the DFE. The gray lines indicate true values, and the data plotted in these figures can be found in supplementary tables S4–S6, Supplementary Material online. (*A*) In this case, simpler demographic models with instantaneous growth or symmetric migration were fit to the neutral data. The resulting misspecified model was then used when inferring the DFE. This misspecification biased *μ* and *σ*, but not *w*. (*B*) In this case, selected data were simulated assuming dominant or recessive mutations, but the DFE was inferred assuming no dominance (*h *=* *0.5). Again, *μ* and *σ* are biased, but *w* is not. (*C*) In this case, selected data were simulated using a mixture of gamma distributions. When these data were fit using our mixture of lognormal distributions, *w* was not biased. (*D*) In this case, selected data were simulated using bivariate lognormal models, with either symmetric or asymmetric marginal distributions. When these data were fit using our symmetric mixture of lognormal distributions, *w* was only slightly biased.

The demographic model is a key assumption of our joint DFE inference procedure. To test how imperfect modeling of demographic history would bias our inference, we simulated both neutral and selected data under a demographic model that included divergence, exponential growth in both populations, and asymmetric migration between populations (supplementary fig. S2*B*, Supplementary Material online). We then fit models that either lacked migration or that modeled instantaneous growth and symmetric migration to the neutral data (supplementary fig. S2*C*, Supplementary Material online). We then used these misspecified models to infer the DFE correlation *w* from the selected data. For both misspecified demographic models, although the inferred ***μ*** and ***σ*** were biased, we found that the inferred *w* was not strongly biased, particularly for large correlations (fig. 2*A*).

Dominance is a potential confounding factor when inferring the joint DFE, since dominance influences allele frequencies differently in populations that have and have not undergone a bottleneck (Balick et al. 2015). Typically, mutation fitness effects in diploids are assumed to be additive, corresponding to a dominance coefficient of *h*** **=** **0.5. To test the effects of dominance on our inference, we simulated nonsynonymous frequency spectra with dominance coefficients of *h*** **=** **0.25 and *h*** **=** **0.75 and then optimized joint DFE parameters under the assumption that *h*** **=** **0.5. We found that an incorrect assumption about dominance did not substantially bias the inferred *w*, although it did bias the inferred ***μ*** and ***σ*** (fig. 2*B*).

The probability distribution assumed for the joint DFE is another potential confounding factor. To test how this might bias inference, we first simulated a true mixture model in which the marginal distributions were gamma (eq. 7), rather than lognormal (eq. 6). In this case, we found that inferred *w* was not substantially biased (fig. 2*C*). We also considered fitting the lognormal mixture model (fig. 1*D*) to data simulated under a bivariate lognormal model (fig. 1*C* and eq. 8). In this case, we found that the inferred mixture component *w* was larger than the simulated bivariate lognormal correlation coefficient ***ρ***, although they were similar (fig. 2*D*). The mixture model assumes symmetric marginal distributions between the two populations, but the bivariate lognormal model is more general and permits asymmetric marginal distributions. When we simulated data under a bivariate model with asymmetric means and variances of the marginal distributions, but fit with a symmetric mixture model, we found only slight bias, similar to the symmetric bivariate case (fig. 2*D*).

**Fig. 3.**
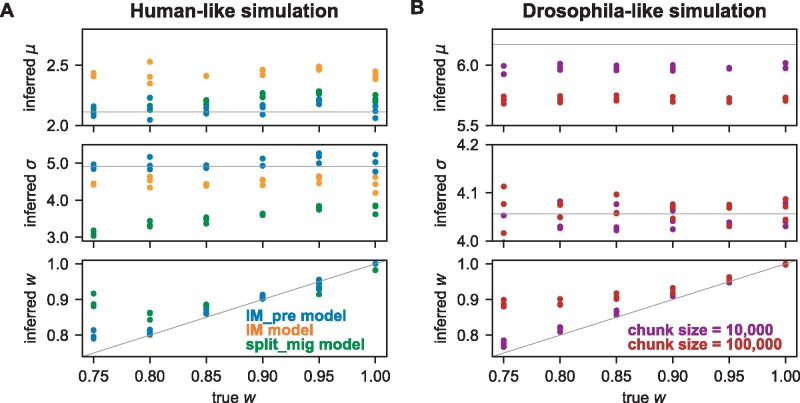
Robustness of joint DFE inference to background selection. Simulated genome-scale data were generated with background selection and different DFE correlations. (*A*) Data were simulated using the best fit demographic model for humans in supplementary figure S6*A*, Supplementary Material online with μ=2.113 and σ=4.915. Beside fitting the true model, simpler demographic models (supplementary fig. S2, Supplementary Material online) were also fit to test robustness to model misspecification in the presence of background selection. (*B*) Data were simulated using the best fit demographic model for *Drosophila melanogaster* in supplementary figure S6*B*, Supplementary Material online with μ=6.174 and σ=4.056. To modulate the strength of background selection, data were simulated with different genomic chunk sizes. The larger chunk size yields stronger background selection. Points indicate inferences from distinct data sets and colors indicate different simulation scenarios. Gray lines indicate true values. The data plotted in these figures can be found in supplementary table S7, Supplementary Material online.

Finally, background selection (BGS) may also bias our joint DFE inference. To examine the effects of BGS on our inference, we simulated data with linkage using SLiM 3 (Haller and Messer 2019). We simulated genome-scale data for both human- and Drosophila-like scenarios using the best fit demographic models we inferred for our real data (supplementary fig. S6*A* and *B*, Supplementary Material online). For each data set, we fit a demographic model to the simulated synonymous mutations then used that demographic model to infer the joint DFE from the simulated nonsynonyous mutations. For human-like simulations, we also carried out the analysis using simpler demographic models in the inference. As expected, we found that BGS biased our demographic model inferences. For example, if we used the same human demographic model in the inference and simulation, the inferred divergence time increased as the DFE correlation *w* decreased (supplementary table S7, Supplementary Material online). As *w* decreased, the strength of BGS increased (supplementary fig. S5 and table S8, Supplementary Material online). However, we found that the joint DFE correlation *w* could be robustly inferred in the presence of BGS (fig. 3). The inferred ***μ*** and ***σ*** were biased if the demographic model was misspecified (fig. 3*A*). But the inferred *w* was overestimated only if *w* was <0.8 with misspecified demographic models (fig. 3*A*). In our Drosophila-like simulations, we simulated under two different regimes to modulate the strength of BGS (supplementary fig. S5, Supplementary Material online). To make those simulations tractable, we scaled *D. melanogaster* population sizes down by a factor of 1,000 and scaled other parameters to attempt to compensate (see Materials and Methods), but rescaling may distort various genetic statistics (Uricchio and Hernandez 2014). Nevertheless, similar to the human simulations, we found bias in the inference of ***μ***, but inference of *w* was biased only if the simulated *w* was <0.9. Because we observed larger values of *w* in our real data analyses, these simulations suggest that those analyses are robust to BGS.

Together, our tests on simulated data suggest that inferring the DFE correlation *w* from the joint AFS can be done with high precision and is robust to multiple confounding factors, including misspecification of the demographic model and DFE distribution as well as the presence of BGS.

### Application

We applied our joint DFE inference approach to humans, *D. melanogaster*, and wild tomatoes. For humans, we considered the joint DFE between Yoruba in Ibadan (YRI) and Utah residents (CEPH) with Northern and Western European ancestry (CEU) populations, because the Yoruba are a well-studied proxy for the ancestral human population and European populations parallel the history of French *D. melanogaster*. For *D. melanogaster*, we considered the joint DFE between Zambian and French populations, because the Zambian population is representative of the ancestral population (Lack et al. 2015) and France is a distinct environment. For wild tomatoes, we considered the joint DFE between two closely related species, *Solanum chilense* and *Solanum peruvianum*, because they still share substantial polymorphism and have overlapping ranges.

We first fit demographic models to synonymous variants in each population pair. For all the three species, we fit relatively simple models of divergence with gene flow, although for humans we also found it necessary to include predivergence population growth. Broadly, these models fit the data well (supplementary figs. S6 and S7, Supplementary Material online).

**Fig. 4.**
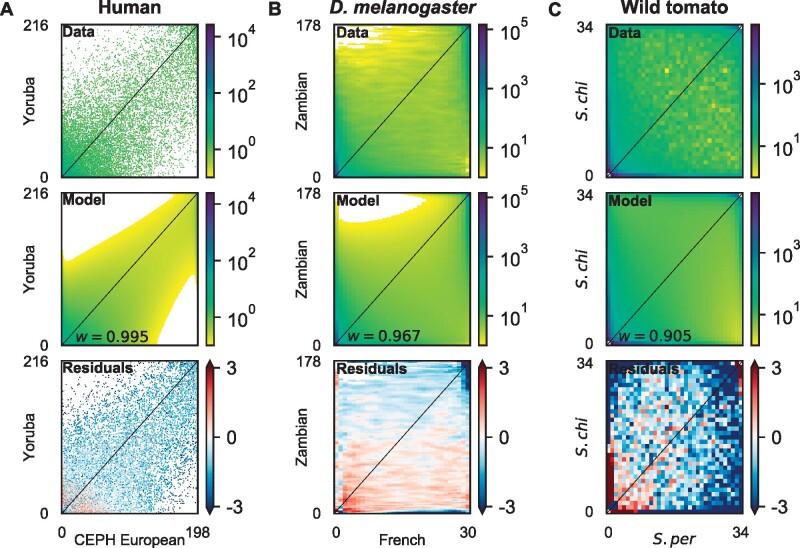
Model fits to joint allele frequency spectra (AFS) using nonsynonymous data. (*A*) Joint AFS for the human nonsynonymous data, the best fit model with DFE correlation *w *=* *0.995, and the residuals between model and data. (*B*) Joint AFS for the *Drosophila melanogaster* nonsynonymous data and the best fit model with DFE correlation *w *=* *0.967. (*C*) Joint AFS for the wild tomato nonsynonymous data and the best fit model with DFE correlation *w *=* *0.905. In all three cases, residuals are small for almost all entries in the AFS, so to increase contrast the color range has been restricted to ±3. See supplementary figure S8, Supplementary Material online for plots showing the full residual range.

**Fig. 5.**
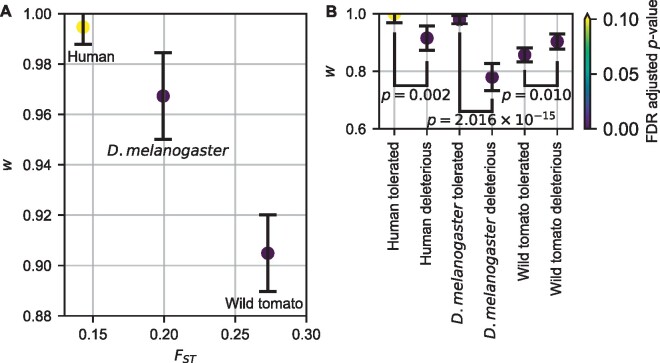
Exome-wide DFE correlations. (*A*) Plotted are maximum likelihood inferences of the DFE correlation *w* with 95% confidence intervals versus genetic divergence *F_ST_* of the considered population pair. (*B*) Plotted are maximum likelihood inferences of the DFE correlation *w* with 95% confidence intervals for nonsynonymous SNPS with different predicted effects from SIFT. Colors indicate FDR adjusted *P*-values from two-tailed *z*-tests as to whether the confidence interval overlaps *w *=* *1. *F_ST_* was estimated using whole-exome synonymous mutations.

We next estimated the joint DFE using all nonsynonymous variants in the whole exome data from each species with our lognormal mixture model (fig. 1*D*). In all the cases, the resulting models fit the nonsynonymous joint frequency spectrum well, with similar patterns of residuals to the demographic models fit to synonymous data (fig. 4 and supplementary fig. S7, Supplementary Material online). For humans, we found the highest DFE correlation w=0.995±0.007 in our study (fig. 5 and supplementary table S9, Supplementary Material online), which was statistically indistinguishable from perfect correlation *w*** **=** **1. For *D. melanogaster*, we found that mutation fitness effects between Zambian and French populations were highly correlated, with w=0.967±0.017 (fig. 5 and supplementary table S10, Supplementary Material online). For wild tomatoes, we found the lowest DFE correlation, w=0.905±0.015 (fig. 5 and supplementary table S11, Supplementary Material online). In humans, CpG dinucleotides have elevated mutation rates, which might affect DFE inference. To control for this effect, we repeated our exome analysis in humans using only regions outside annotated CpG islands. We also did a similar analysis in *D. melanogaster*, although their CpG dinucleotides do not have elevated mutation rates. The resulting estimates of *w* (supplementary fig. S12 and supplementary tables S9, S10, Supplementary Material online) were statistically indistinguishable from those using the whole exome data. We further inferred DFE correlation using only GC-conservative mutations (A **↔** T and C **↔** G) in humans, because GC-biased gene conversion (gBGC), which is common in mammals but not in *D. melanogaster* (Zhen et al. 2021), may bias DFE inference (Castellano et al. 2019). These GC-conservative mutations are not affected by gBGC. Similar to the whole exome data, the resulting *w* was statistically indistinguishable from 1 (supplementary fig. S12 and table S9, Supplementary Material online). Among these three population pairs, the inferred DFE correlation was negatively related to genetic divergence, as measured by *F_ST_* (fig. 5*A*).

For simplicity, we assumed that the DFE correlation *w* is constant throughout the distribution, but the correlation may depend on how deleterious the mutation is. To test this assumption, rather than adding complexity to the DFE model, we instead segregated our data by applying SIFT scores to predict whether a nonsynonymous mutation is likely to be tolerated or deleterious based on evolutionary conservation (Vaser et al. 2016). We then fit DFE models to the SNPs in each class. As expected, we inferred a more negative mean fitness effect for the deleterious class than the tolerated class (supplementary fig. S12 and tables S9–S11, Supplementary Material online). Moreover, we found that the DFE correlation *w* was dramatically smaller for the deleterious class than the value from the tolerated class in humans and *D. melanogaster*, but not in wild tomatoes (fig. 5*B*). To test whether this effect extended beyond individual mutations to whole genes, we also separated our data by the d*N*/d*S* ratio in humans and *D. melanogaster*. We found no significant difference in DFE correlations among genes with different d*N*/d*S* ratios (supplementary fig. S12, Supplementary Material online). However, we did observe that the average strength of purifying selection increases as the d*N*/d*S* ratio decreases (supplementary fig. S12, Supplementary Material online).

**Fig. 6.**
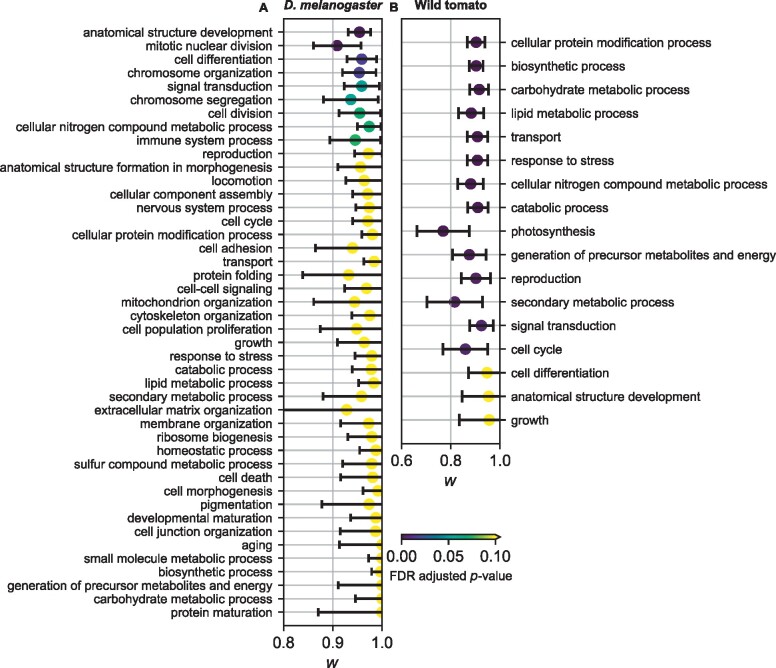
DFE correlation for different GO terms in *Drosophila melanogaster* and wild tomatoes. Plotted are maximum likelihood inferences with 95% confidence intervals. Colors indicate FDR-adjusted *P*-values from two-tailed *z*-tests as to whether the confidence interval overlaps *w *=* *1. The data plotted in these figures can be found in supplementary tables S10 and S11, Supplementary Material online. (*A*) Inferred DFE correlation in *D. melanogaster*. (*B*) Inferred DFE correlation in wild tomatoes.

To investigate the biological basis of the joint DFE, we considered genes of different function based on Gene Ontology (GO) terms (Gene Ontology Consortium 2000). For *D. melanogaster*, we found a wide range of inferred DFE correlations, with the lowest maximum likelihood estimate corresponding to mutations in genes involved in the mitotic nuclear division at w=0.901±0.048 (fig. 6 and supplementary table S10, Supplementary Material online). For wild tomatoes, we found an even wider range of inferred DFE correlations, with the lowest maximum likelihood estimate being genes involved in photosynthesis at w=0.769±0.106 (fig. 6 and supplementary table S11, Supplementary Material online). For humans, we found that all GO terms yielded values of *w* that were statistically indistinguishable from one (supplementary table S9 and fig. S9, Supplementary Material online). Among the *D. melanogaster* GO terms, we found no correlation between the inferred *w* and the mean and standard deviation of the marginal DFEs (supplementary fig. S13, Supplementary Material online), suggesting that the variation we see in *w* is not driven simply by variation in overall constraint. In humans, we further explored the biological context of the joint DFE by considering genes that are involved in disease and that interact with viral pathogens. We found no statistically significant differences in DFE correlations among these gene groups, although we did find that the DFE for genes involved in disease or that interact with viruses was shifted toward more negative selection (supplementary table S9 and fig. S12, Supplementary Material online).

To test the robustness of our analyses in the real data to various modeling choices, we used the variation among our inferences among *D. melanogaster* GO terms. We fit simpler models of demographic history with instantaneous growth in the two diverged populations with and without symmetric migration to the synonymous data and used those models as the basis of joint DFE analysis. Although these demographic models fit the data much less well than our main model (supplementary figs. S7 and S14, Supplementary Material online), the inferred values of *w* for the GO terms were highly correlated with those from our main model (supplementary fig. S15*A* and *B*, Supplementary Material online). We also tested our approach using a DFE model with a bivariate lognormal model instead of a lognormal mixture model. The inferred values for ***ρ*** in the bivariate model were highly correlated with the values for the inferred *w* (supplementary fig. S15*C*, Supplementary Material online). Together, these results suggest that the robustness we observed in simulated data (fig. 2) holds true for real data.

## Discussion

In this study, we introduced the concept of a joint DFE between pairs of populations, and we developed and applied an approach for inferring it. We tested our approach with simulation studies and found that inferring the DFE correlation between populations does not require excessive data and is robust to many forms of model misspecification (supplementary fig. S3, Supplementary Material online and figs. 2 and 3). We then applied our approach to humans, *D. melanogaster*, and wild tomatoes. Among these species, we found the lowest exome-wide DFE correlation in wild tomatoes and the highest in humans (fig. 5*A*). In humans and *D. melanogaster*, we found that the DFE correlation is lower for deleterious mutations than tolerated mutations (fig. 5*B*). And in *D. melanogaster* and tomatoes, we found that the DFE correlation varied with gene function (fig. 6). These results illustrate the biological insights that can be gained by considering the joint DFE between populations.

The first step of our analyses is fitting a demographic model, although our DFE correlation inferences are robust to details of that model (fig. 2*A* and supplementary fig. S14, Supplementary Material online). Nevertheless, our inferred demographic models (supplementary fig. S6, Supplementary Material online) are comparable to other inferences. For *D. melanogaster*, our inferred relative population sizes and divergence time for African and European populations are similar to those of Arguello et al. (2019) (supplementary table S16, Supplementary Material online), although we used different populations and different models. For humans, our demographic parameters were similar to those of Gravel et al. (2011) (supplementary table S17, Supplementary Material online), although their model also included an East Asian population. For wild tomatoes, we obtained a demographic model close to the result of Beddows et al. (2017) (supplementary table S18, Supplementary Material online).

The fitness effect of a mutation may differ between populations due to differences in both environmental and genetic context. The wild tomato species we analyzed overlap in range and are more genetically differentiated than the *D. melanogaster* or human populations we studied. In this case, we speculate that differences in fitness effects are primarily driven by differences in genetic background, although *S. chilense* does exhibit adaptations for more arid habitats (Moyle 2008). Among the species we studied, humans exhibited the highest correlation of mutation fitness effects, which was statistically indistinguishable from perfect correlation *w*** **=** **1, suggesting little difference in mutation fitness effects between YRI and CEU populations. Huang et al. (2021) also estimated the genome-wide differences of selection coefficients between Africans and Europeans were almost 0 with a different approach (He et al. 2015). It is unclear whether this is caused by our relatively low genetic differentiation or our ability to control our local environment. Experiments suggest that stressful environments can alter DFEs between populations (Wang et al. 2014). Previous population genetic studies also have found evidence for differences in marginal DFEs between populations of humans (Boyko et al. 2008; Lopez et al. 2018) and also between populations of other primates (Ma et al. 2013; Castellano et al. 2019; Tataru and Bataillon 2019). Although we assumed that the mean and the variance of mutation fitness effects did not differ between the two populations in our models for the joint DFE, those previous studies found only slight differences and our simulation study suggests that inferences of the DFE correlation are robust to relatively large differences in marginal DFEs (fig. 2*D*). Recently, Martin and Lenormand (2015) extended Fisher’s Geometrical Model to consider the relationship between mutation fitness effects in two different environments, represented by two optima in trait space. Unfortunately, they could not derive an analytic joint DFE for their model, so we could not apply it here. In related work, Keightley et al. (2000) used *Caenorhabditis elegans* mutation accumulation data to infer bivariate gamma distributions of mutation effects on pairs of life history traits, although with low precision. Overall, our simple models of the joint DFE fit the data well, but more complex models may be more informative. Over the long term, assessing the joint DFE between multiple populations of multiple species may reveal the relative importance of environmental and genetic context in determining the mutation fitness effects.

We focused on the deleterious component of the DFE in this study, and positive selection or local adaptation may affect joint DFE inference. However, Castellano et al. (2019) found that including beneficial mutations or not did not affect the DFE model for the deleterious components in humans. Moreover, Zhen et al. (2021) estimated the proportion of new beneficial mutations to be ∼1.5% in humans and close to 0 in *D. melanogaster*. Therefore, we do not expect beneficial mutations to significantly affect our inference in humans and *D. melanogaster*. Further studies that include local adaptation when inferring the joint DFE may improve our analysis of populations with low DFE correlations, such as wild tomatoes.

Finally, the concept of a joint DFE could be widely applicable. For example, we recently inferred a joint DFE between mutations at the same protein site, using triallelic variants (Ragsdale et al. 2016). Remarkably, we found that biochemical experiments in a variety of organisms yielded a similar correlation of pairwise fitness effects to the value we inferred from *D. melanogaster* population genetic data. Other potential applications of a joint DFE include modeling ancient DNA data to infer DFE correlations across time and modeling linkage to infer DFE correlations across genomic positions. We thus anticipate that extending the concept of the DFE from one population to two or more will significantly advance our understanding of population evolution and have broad impact in population genetics.

## Materials and Methods

### Inferring the Joint DFE from the Joint AFS

If we sample *n*_1_ chromosomes from population 1 and *n*_2_ chromosomes from population 2, then the joint AFS for these two populations can be written a*s*

(1) $$X=\left\{X_{i,j},0\leq i\leq n_{1},0\leq j\leq n_{2},0<i+j<n_{1}+n_{2}\right\}.$$

Here, $X_{i,j}$ denotes the number of mutations in the sample that have *i* copies of derived alleles among the *n*_1_ chromosomes from population 1 and *j* copies of derived alleles among the *n*_2_ chromosomes from population 2. We denote the joint spectra for neutral and selected mutations as $N=\left\{N_{i,j}\right\}$ and $S=\left\{S_{i,j}\right\}$, respectively.

Let $F(\gamma_{1},\gamma_{2}|\Theta_{\text{demo}})=\left\{F_{i,j}(\gamma_{1},\gamma_{2}|\Theta_{\text{demo}})\right\}$ be the expected joint AFS for demographic parameters $\Theta_{\text{demo}}$, population-scaled selection coefficients ***γ***_1_ in the ancestral and first contemporary population and ***γ***_2_ in the second contemporary population, and population-scaled mutation rate ***θ***= 1. The population-scaled selection coefficient ***γ*** is $2N_{a}s$, where *N_a_* is the ancestral population size. For a mutation with selection coefficient *s*, a diploid individual has its fitness multiplied by 1+2s if homozygous and by 1+2hs if heterozygous, where *h* is the dominance coefficient. The population-scaled mutation rate ***θ*** is $4N_{a}\mu$, where ***μ*** is the mutation rate. The vector of demographic parameters $\Theta_{\text{demo}}$ depends on the demographic model assumed, but it typically contains parameters for relative population sizes, divergence times, and rates of gene flow. Then the expected neutral joint AFS is

(2) $$E\left(N_{i,j}|\Theta_{\text{demo}}\right)=\theta_{\text{neu}}F_{i,j}(\gamma_{1}=0,\gamma_{2}=0|\Theta_{\text{demo}}),$$

where $\theta_{\text{neu}}$ is the population-scaled neutral mutation rate (Gutenkunst et al. 2009). The expected selected joint AFS i*s*

(3) $$E\left(S_{i,j}|\Theta_{\text{demo}},\Theta_{\text{DFE}}\right)=\theta_{\text{sel}}\int_{-\infty }^{\infty }\int_{-\infty }^{\infty }F_{i,j}(\gamma_{1},\gamma_{2}|\Theta_{\text{demo}})G(\gamma_{1},\gamma_{2}|\Theta_{\text{DFE}})d\gamma_{1}d\gamma_{2}.$$

Here $\theta_{\text{sel}}$ is the population-scaled mutation rate for selected mutations, and $G(\gamma_{1},\gamma_{2}|\Theta_{\text{DFE}})$ is the joint DFE.

In most of our analyses, we modeled the joint DFE as a mixture of two components, $G_{1d}$ and $G_{2d}$, where $G_{1d}$ is a DFE with equal selection coefficients in the two populations, and $G_{2d}$ is a DFE with statistically independent selection coefficients and marginal distributions $G_{1d}$. Letting *w* be the mixture proportion of $G_{1d}$, we have

(4) $$G_{\text{mix}}=wG_{1d}+\left(1-w\right)G_{2d},\;0\leq w\leq 1.$$

And considering only deleterious mutations we have

(5) $$\begin{array}{c} E\left(S_{i,j}|\Theta_{\text{demo}},\Theta_{\text{DFE}}\right)=w\theta_{\text{sel}}\int_{-\infty }^{0}F_{i,j}(\gamma ,\gamma |\Theta_{\text{demo}})G_{1d}(\gamma |\Theta_{\text{DFE}})d\gamma \\ +(1-w)\theta_{\text{sel}}\int_{-\infty }^{0}\int_{-\infty }^{0}F_{i,j}(\gamma_{1},\gamma_{2}|\Theta_{\text{demo}})G_{2d}(\gamma_{1},\gamma_{2}|\Theta_{\text{DFE}})d\gamma_{1}d\gamma_{2}. \end{array}$$

We typically worked with lognormal distributions, so

(6) $$\begin{array}{c} G_{1d}(\gamma )=\frac{1}{\gamma \sigma \sqrt{2\pi }}\;\;\text{exp}\;\left(-\frac{{\left(\ln(-\gamma )-\mu \right)}^{2}}{2\sigma^{2}}\right),\\ G_{2d}(\gamma_{1},\gamma_{2})=\frac{1}{\gamma_{1}\gamma_{2}\sigma^{2}2\pi }\;\;\text{exp}\;\left(-\frac{{\left(\ln(-\gamma_{1})-\mu \right)}^{2}+{\left(\ln(-\gamma_{2})-\mu \right)}^{2}}{2\sigma^{2}}\right). \end{array}$$

Here, ***μ*** and ***σ*** are the mean and standard deviation of the logs of the population-scaled selection coefficients, respectively.

To test the robustness of our approach, we also considered other models for the joint DFE. When using a mixture of gamma distributions,

(7) $$\begin{array}{c} G_{1d}(\gamma )=\frac{1}{\beta^{\alpha }\Gamma (\alpha )}{\left(-\gamma \right)}^{\alpha -1}\;\text{exp}(\gamma /\beta )\\ G_{2d}(\gamma_{1},\gamma_{2})=\frac{1}{\beta^{2\alpha }\Gamma {\left(\alpha \right)}^{2}}{\left(\gamma_{1}\gamma_{2}\right)}^{\alpha -1}\;\text{exp}((\gamma_{1}+\gamma_{2})/\beta ). \end{array}$$

Here, ***α*** is the shape parameter and ***β*** is the scale parameter. When using a bivariate lognormal distribution, which is potentially asymmetric,

(8) $$\begin{array}{c} G(\gamma_{1},\gamma_{2})=\frac{1}{2\pi \sigma_{1}\sigma_{2}\gamma_{1}\gamma_{2}\sqrt{1-\rho^{2}}}\\ \times \text{exp}\;\left(-\frac{1}{2}\left(\frac{{\left(\ln(-\gamma_{1})-\mu_{1}\right)}^{2}}{\sigma_{1}^{2}}+\frac{{\left(\ln(-\gamma_{2})-\mu_{2}\right)}^{2}}{\sigma_{2}^{2}}-\frac{2\rho \left(\ln(-\gamma_{1})-\mu_{1}\right)\left(\ln(-\gamma_{2})-\mu_{2}\right)}{\sigma_{1}\sigma_{2}}\right)\right). \end{array}$$

Here, ***ρ*** is the correlation coefficient.

Calculating the expected selected joint AFS (eqs. 3 and 5) is computationally expensive, because spectra $F(\gamma_{1},\gamma_{2}|\Theta_{\text{demo}})$ must be calculated for many pairs of selection coefficients. Simultaneously inferring the demographic parameters $\Theta_{\text{demo}}$ and the DFE parameters $\Theta_{\text{DFE}}$ is thus infeasible. We thus first inferred the demographic parameters using the putative neutral data and then held those parameters constant while inferring the DFE parameters.

Ancestral state misidentification creates an excess of high-frequency derived alleles (Baudry and Depaulis 2003), which may bias demographic history and DFE inference. To account for this effect, when fitting demographic history and DFE models we included separate parameters $p_{\text{misid}}$ for ancestral state misidentification (Ragsdale et al. 2016). Then, for example,

(9) $$E(N_{i,j}|\Theta_{\text{demo}},p_{\text{Nmisid}})=(1-p_{\text{Nmisid}})E(N_{i,j}|\Theta_{\text{demo}})+p_{\text{Nmisid}}E(N_{n_{1}-i,n_{2}-j}|\Theta_{\text{demo}}).$$

when ancestral state misidentification is applied to the neutral demographic history model.

We inferred the demographic parameters ${\hat{\Theta }}_{\text{demo}}$ by maximizing the composite likelihood of the neutral joint AFS, including $\theta_{\text{neu}}$ as a free parameter (Gutenkunst et al. 2009). To then infer the DFE parameters $\Theta_{\text{DFE}}$, we modeled the selected joint AFS as a Poisson Random Field (Sawyer and Hartl 1992) and maximized the composite likelihood

(10) $$\ell (S|{\hat{\Theta }}_{\text{demo}},\Theta_{\text{DFE}},p_{\text{Smisid}})=\prod_{i,j}\frac{\;\text{exp}[-E(S_{i,j}|{\hat{\Theta }}_{\text{demo}},\Theta_{\text{DFE}},p_{\text{Smisid}})]E{\left(S_{i,j}|{\hat{\Theta }}_{\text{demo}},\Theta_{\text{DFE}},p_{\text{Smisid}}\right)}^{S_{i,j}}}{S_{i,j}!}.$$

Here, ${\hat{\Theta }}_{\text{demo}}$ represents the demographic parameters inferred from the neutral data. And in this step we fixed $\theta_{\text{sel}}$ to a multiple of $\theta_{\text{neu}}$ determined by the expected ratio of new selected to new neutral mutations, based on base-specific mutation rates and genome composition.

Numerically, to calculate the expected selected joint AFS, we first cached expected spectra $F(\gamma_{1},\gamma_{2}|{\hat{\Theta }}_{\text{DFE}})$ for a range of selection coefficient pairs. The cached values of ***γ***_1_, ***γ***_2_ were from 50 points logarithmically spaced within $\left[-10^{-4},-2000\right]$, for a total of 2,500 cached spectra (supplementary fig. S1, Supplementary Material online). We then evaluated equation (3) using the trapezoid rule over these cached points. To test whether the accuracy of this integration affected our results, we repeated our exome-wide analyses for humans and *D. melanogaster* using 100 cache points, for a total of 10,000 cached spectra. The results of these analyses were statistically indistinguishable from those using 50 cache points (supplementary table S13, Supplementary Material online). For the mixture model (eq. 5), the $G_{1d}$ component was calculated as a one-dimensional integral over a cache of $\gamma_{1}=\gamma_{2}$ spectra. Probability density for the joint DFE may extend outside the range of cached spectra. To account for this density, we integrated outward from the sampled domain to ***γ***= 0 or −∞ to estimate the excluded weight of the joint DFE. We then weighted the closest cached joint AFS **F** by the result and added it to the expected joint AFS. For the edges of the domain, this was done using the SciPy method quad, and for the corners it was done using dblquad (Virtanen et al. 2020).

### Simulated Data

For our precision tests (supplementary fig. S3, Supplementary Material online), we used dadi to simulate data sets without linkage. Unless otherwise specified, for supplementary figure S3, Supplementary Material online and figure 2, the “truth” simulations were performed with an isolation-with-migration (IM) demographic model (supplementary fig. S2*B*, Supplementary Material online) with parameters as in supplementary table S1, Supplementary Material online, a joint lognormal mixture DFE model with marginal mean μ=3.6 and standard deviation σ=5.1, and with sample sizes of 216 for population 1 and 198 for population 2. For supplementary figure S3, Supplementary Material online, data were simulated with *w*** **=** **0.9 and the nonsynonymous population-scaled mutation rate $\theta_{NS}=13842.5$ by Poisson sampling from the expected joint AFS. For supplementary figure S3*A*, Supplementary Material online, the resulting average number of segregating polymorphisms varied with sample size, ranging from 6,953 for sampling two chromosomes to 45,691 for sampling 100 chromosomes. For supplementary figure S3*B* and *C*, Supplementary Material online, the sample size was fixed at 20 chromosomes per population.

For our robustness tests (fig. 2), we were interested in bias rather than variance, so misspecified models were fit directly to the expected frequency spectrum under the true model without Poisson sampling noise. For figure 2*A*, the best fit model with no migration had *s*** **=** **0.937, $\nu_{1}=3.025,\;\nu_{2}=3.219$, *T*** **=** **0.0639, *m*** **=** **0, and the best fit model with instantaneous growth and symmetric migration had $\nu_{1}=2.4,\;\nu_{2}=0.92$, *T*** **=** **0.23, *m*** **=** **0.42. For figure 2*C*, the true joint DFE was a mixture model with marginal gamma distributions with α=0.4, ***β* **= 1400. For figure 2*D*, the true joint DFE was a symmetric bivariate lognormal distribution with μ=3.6 and σ=5.1, and for the asymmetric case in figure 2*D*, $\mu_{1}=3.6,\;\sigma_{1}=5.1,\;\mu_{2}=4.5,\;\sigma_{2}=6.8$. We then simulated data with different correlation coefficients ***ρ*** to examine the relationship between ***ρ*** and the DFE correlation *w*.

To examine the effects of BGS, we used SLiM 3 (Haller and Messer 2019) to simulate data with linkage. We replicated our simulation and inference three times for each *w* with different demographic models in the human simulations and an IM model in the *D. melanogaster* simulations (supplementary fig. S2, Supplementary Material online). For humans, we simulated the exome in chromosome 21 using the demographic parameters in supplementary figure S6*A*, Supplementary Material online, the joint DFE parameters ***μ*** and ***σ*** from the whole human exome in supplementary table S9, Supplementary Material online with w=0.75,0.8,0.85,0.9,0.95,1, and sample sizes of 216 for population 1 and 198 for population 2. We assumed the mutation rate was $1.5\times 10^{-8}$ per nucleotide per generation ( Ségurel et al. 2014) and an ancestral population size of 8,000. We further assumed the ratio of the nonsynonymous to synonymous mutations in humans was 2.31 (Huber et al. 2017). In our simulation, we used the human exome based on the reference genome hg19 from UCSC Genome Browser and the deCODE human genetic map (Kong et al. 2010). For each *w*, we first simulated human chromosome 21 twenty times, then obtained 20 synonymous frequency spectra and 20 nonsynonymous frequency spectra from these sequences. We combined these 20 synonymous frequency spectra into a single one and inferred the demographic models. We then combined the 20 nonsynoymous frequency spectra into one spectrum and inferred the joint DFEs. We inferred the joint DFEs using both the true (IM_pre model) and wrong (IM model with asymmetric migration & split_mig model without migration) demographic models (supplementary fig. S2, Supplementary Material online). For *D. melanogaster*, we simulated small sequences instead of a whole chromosome, because the large population size of *D. melanogaster* made our simulation extremely slow. We used the demographic parameters for the IM model in supplementary figure S6*B*, Supplementary Material online, the joint DFE parameters ***μ*** and ***σ*** from the whole *D. melanogaster* exome in supplementary table S10, Supplementary Material online with w=0.75,0.8,0.85,0.9,0.95,1, and sample sizes of 178 for population 1 and 30 for population 2. For each *w*, we simulated 2000 small sequences with a length of 10,000 bp, then obtained 2,000 synonymous frequency spectra and 2,000 nonsynonymous frequency spectra. We combined these 2,000 synonymous frequency spectra into a single one and inferred the demographic models. We then combined the 2,000 nonsynonymous frequency spectra into one spectrum and inferred the joint DFEs. This was equivalent to a total sequence size of 20 Mb. We also replicated the above simulation with 200 small sequences with a length of 100,000 bp. We inferred the demographic and DFE parameters from the combined synonymous frequency spectrum and nonsynonymous frequency spectrum of these 200 small sequences. We assumed that the mutation rate was $2.8\times 10^{-9}$ per nucleotide per generation, that the recombination rate was $5\times 10^{-9}$ per nucleotide per generation (Keightley et al. 2014), and that the ancestral population size was 1.38 million. We also assumed the ratio of the nonsynonymous to synonymous mutations in *D. melanogaster* was 2.85 (Huber et al. 2017). For *D. melanogaster*, to accelerate our simulation, we used a factor of 1,000 to rescale the population size, mutation rate, and recombination rate (Hoggart et al. 2007). To quantify the strength of BGS in our simulations, we simulated data under neutral models and compared the expected number of pairwise differences between two chromosomes in the nonneutral scenarios with the neutral ones (Hudson and Kaplan 1995). The strength of BGS (supplementary fig. S5, Supplementary Material online) in the simulated data for both humans and *D. melanogaster* was comparable to or stronger than the estimated strength from the empirical studies (Charlesworth 2013).

### Genomic Data

In all analyses, we only considered biallelic SNPs from automosomes. For humans, we obtained 108 and 99 unrelated individuals (216 and 198 haplotypes) from YRI and CEU populations in the 1000 Genomes Project Phase 3 genotype data (1000 Genomes Project Consortium 2015). We removed those regions that were not in the 1000 Genomes Project phase 3 strict mask file. We only considered biallelic exonic SNPs that were annotated as synonymous_variant or missense_variant by the 1000 Genomes Project. We further excluded SNPs without reported ancestral alleles. We also used the CpG table from the UCSC Genome Browser to distinguish SNPs in CpG regions. We further used mutations unaffected by gBGC (only A **↔** T and C **↔** G mutations) to repeat our analysis.

For *D. melanogaster*, we obtained Zambian and French *D. melanogaster* genomic data from the Drosophila Genome Nexus (Lack et al. 2016). The Zambian sequences were 197 haploids from the DPGP3 and the French were 87 inbred individuals. We removed those SNPs in the IBD and/or admixture masks. In these data, many SNPs were not called in all individuals. We thus projected downward to obtain a consensus AFS with maximal genome coverage. For these data, that was to a sample size of 178 Zambian and 30 French haplotypes (supplementary fig. S16, Supplementary Material online). We used *Drosophila simulans* as the outgroup and downloaded the alignment between the reference genome for *D. simulans* (drosim1) and the reference genome for *D. melanogaster* (dm3) from UCSC Genome Browser to determine the ancestral allele of each SNP. We then used GATK (version: 4.1.4.1) (McKenna et al. 2010) to liftover the genomic coordinates from dm3 to dm6 with the liftover chain file from the UCSC Genome Browser. To annotate SNPs to their corresponding genes and as synonymous or nonsynonymous mutations, we used ANNOVAR (version: 20191024) (Wang et al. 2010) with default settings and the dm6 genome build. We downloaded the CpG table from the UCSC Genome Browser to distinguish SNPs in CpG regions.

For wild tomatoes, we obtained *S. chilense* and *S. peruvianum* DNA sequencing data from Beddows et al. (2017) and followed their scheme for assigning individuals to species. We only analyzed 17 *S. chilense* and 17 *S. peruvianum* individuals sequenced by Beddows et al. (2017) because of their high quality. We used an *Solanum lycopersicoides* individual sequenced by Beddows et al. (2017) to determine the ancestral allele of each SNP. We further removed variants with heterozygous genotype in this *S. lycopersicoides* individual. To more easily apply SIFT, we used the NCBI genome remapping service to convert the data from SL2.50 coordinates to SL2.40.

### Fitting Demographic Models to Genomic Data

We used dadi to fit models for demography to spectra for synonymous mutations (Gutenkunst et al. 2009), including a parameter for ancestral state misidentification (Ragsdale et al. 2016). For the human analysis, we used dadi with grid points of [226,236,246], and we found that an IM model with an instantaneous growth in the ancestral population (IM_pre) fit the data well (supplementary fig. S6*A*, Supplementary Material online). For the *D. melanogaster* analysis, we used dadi with grid points of [188,198,208], and we found that an IM model fit the data well (supplementary fig. S6*B*, Supplementary Material online). For the wild tomato analysis, we used dadi with grid points of [44,54,64] and fit a split-migration model with asymmetric migration (supplementary fig. S6*C*, Supplementary Material online), as Beddows et al. (2017) did.

### Fitting Joint DFEs to Genomic Data

Cached allele frequency spectra were created for the corresponding demographic models. For humans and *D. melanogaster*, we used the same grid points settings as the grid points used when inferring demographic models. For wild tomatoes, we used dadi with grid points of [300,400,500] to generate caches with selection. Models of the joint DFE were then fit to nonsynonymous data by maximizing the likelihood of the data, assuming a Poisson Random Field (Sawyer and Hartl 1992). In these fits, the population-scaled mutation rate for nonsynonymous mutations ***θ***_*NS*_ was held fixed at a given ratio to the rate for synonymous mutations ***θ***_*S*_ in the same subset of genes, as inferred from our demographic history model. For *D. melanogaster* this ratio was 2.85 and for humans it was 2.31 (Huber et al. 2017). This ratio was 5.21 for the gBGC unaffected mutations in humans (Zhen et al. 2021). For wild tomatoes, this ratio was assumed to be 2.5, which was between the ratios in humans and *D. melanogaster*. For the lognormal mixture model, the three parameters of interest are the DFE correlation *w* as well as the mean ***μ*** and standard deviation ***σ*** of the marginal distributions. In addition, we included a separate parameter for ancestral state misidentification for each subset of the data tested, because the rate of misidentification depends on the strength of selection acting on the sites of interest. To mitigate the effect of BGS, we separately inferred demographic parameters for each subset of the data (supplementary tables S9–S11, Supplementary Material online) with the best fit demographic model inferred from the whole exome data (supplementary fig. S6, Supplementary Material online).

We separately analyzed SNPs from genes associated with different GO terms. We downloaded the Generic GO subset from http://geneontology.org/docs/download-ontology/ on August 12, 2020. This is a set of curated terms that are applicable to a range of species (Gene Ontology Consortium 2000). We considered the direct children of GO: 0008510 “Biological Process,” and any gene annotated with a child of a given term was assumed to also be annotated by the parent term. Thus, a given gene may be present in multiple GO terms in our analysis. We used Ensembl Biomart (Cunningham et al. 2019) to retrieve the annotated GO terms for each gene. For humans, we downloaded the GO annotation from https://grch37.ensembl.org/biomart/martview/ with Ensembl Genes 101 database and Human genes (GRCh37.p13) on August 19, 2020. For *D. melanogaster*, we downloaded the GO annotation from https://www.ensembl.org/biomart/martview/ with Ensembl Genes 101 database and *D. melanogaster* genes (BDGP6.28) on September 10, 2020. For tomatoes, we downloaded the GO annotation from https://jul2018-plants.ensembl.org/biomart/martview/ with Ensembl Plants Genes 40 database and Solanum lycopersicum genes (SL2.50) on September 26, 2020. To ensure convergence in our inference, we removed those GO terms with <2,000 either synonymous or nonsynonymous mutations (supplementary tables S9–S11, Supplementary Material online).

We also separately analyzed SNPs classified by SIFT as deleterious (SIFT score ≤ 0.05) or tolerated (SIFT score > 0.05) (Vaser et al. 2016). We downloaded SIFT predictions from https://sift.bii.a-star.edu.sg/sift4g/ on October 2, 2020. We used the SIFT prediction data with GRCH37.74 for humans, with BDGP6.83 for *D. melanogaster*, and with SL2.40.26 for tomatoes. To carry out our DFE analysis, we needed to estimate an appropriate population-scaled nonsynonymous mutation rate ***θ***_*NS*_ for deleterious and tolerated mutations. To do so, we estimated the proportions of deleterious and tolerated mutations in the downloaded SIFT prediction data sets. This is because all the possible mutations and their SIFT scores were predicted in the downloaded data sets. We then obtained the population-scaled mutation rates for deleterious and tolerated mutations by multiplying ***θ***_*NS*_ from the whole exome data with the proportions of deleterious and tolerated mutations, respectively. More specifically, if we assumed the mutation rate for the *i*th type nucleotide mutation to be *u_i_*, the count for deleterious mutations from the *i*th type nucleotide mutation to be *d_i_* in the SIFT data sets, and the count for tolerated mutations from the *i*th type nucleotide mutation to be *t_i_* in the SIFT data sets, then the proportions for deleterious and tolerated mutations were $\sum_{i}u_{i}d_{i}/[\sum_{i}u_{i}\sum_{i}\left(d_{i}+t_{i}\right)]$ and $\sum_{i}u_{i}t_{i}/[\sum_{i}u_{i}\sum_{i}\left(d_{i}+t_{i}\right)]$, respectively. In total, we have 12 different types of nucleotide mutations: A **→** T, T **→** A, C **→** G, G **→** C, A **→** C, C **→** A, C **→** T, T **→** C, A **→** G, G **→** A, G **→** T, and T **→** G. We obtained the mutation rates for different types of mutations from Jónsson et al. (2017) for humans and Singh et al. (2007) for drosophila. For wild tomatoes, we used the *Arabidopsis thaliana* nucleotide mutation rates from Ossowski et al. (2010), because these mutation rates have not been directly measured in wild tomatoes.

We further considered differences between regions of the genome that experience different levels of evolutionary conservation, as estimated from the ratio of nonsynonymous to synonymous divergence d*N*/d*S*. For humans, we separated SNPs into categories based on the estimated d*N*/d*S* values of the gene in which they are found from a previous study (Gayà-Vidal and Albà 2014). For *D. melanogaster*, we separated SNPs based on the d*N*/d*S* estimate of the surrounding 10 kb genomic region from PopFly (Hervas et al. 2017).

For humans, we also divided genes into classes based on their role in disease and interactions with viruses. Following Struck et al. (2018), we classified genes as associated with Mendelian disease, complex disease, or no disease using Online Mendelian Inheritance in Man (OMIM, Amberger et al. 2015) and the European Bioinformatics Institute’s genome-wide association studies (GWAS) catalog (MacArthur et al. 2017). We used the data of Enard and Petrov (2018) to annotate 4,534 genes as encoding virus-interacting proteins (VIPs). We defined the set of non-VIP genes as the 17,603 Ensembl genes that were not annotated as encoding VIPs. We identified 1,728 genes as known to interact with 2 or more viruses, leaving 2,806 genes known to interact with only a single virus.

To estimate the uncertainty of our inferences, we used an approach based on the Godambe Information Matrix (Coffman et al. 2016), which is computationally more efficient than conventional bootstrap parameter optimization. To generate the requisite bootstrap data sets, we divided the reference genomes into 1 Mb chunks. Because gene content varied among bootstraps, ***θ***_*NS*_ also needed to vary. To estimate the appropriate ***θ***_*NS*_ for each bootstrap, we scaled corresponding ***θ***_*NS*_ from the real data by the ratio of the number of segregating sites in the AFS of the bootstrap versus real data. We found good agreement between the uncertainties estimated by the Godambe approach and those from directly fitting the bootstrap data sets (supplementary fig. S17, Supplementary Material online). Note that this process does not propagate uncertainty in the demographic parameter inference, so our uncertainties are somewhat underestimated.

To estimate *P*-values for inferred DFE correlation *w*, we used the two-tailed *z*-test by assuming *w*** **=** **1 under the null hypothesis and using the standard deviation estimated from the Godambe approach. To compare inferred DFE correlations between tolerated and deleterious mutations, we used two-tailed *z*-tests to calculate *P*-values by assuming no difference under the null hypothesis and using the standard deviations estimated from the Godambe approach. For multiple testing correction, we estimated false discovery rate (FDR) adjusted *P*-values by the Benjamini–Hochberg procedure (Benjamini and Hochberg 1995). These multiple hypothesis tests are from different types of data, including whole-exome data, whole-exome data without CpG regions, different GO terms, genes with different d*N*/d*S* values, genes with different SIFT scores, genes associated with no/simple/complex diseases, and genes associated with no/single/multiple VIPs (supplementary tables S9–S11, Supplementary Material online).

## Supplementary Material

Supplementary data are available at *Molecular Biology and Evolution* online.

## Supplementary Material

msab162_Supplementary_Data
