# Supplementary material for: Inferring Genome-Wide Correlations of Mutation Fitness Effects between Populations
Source: Mol Biol Evol. 2021 May 27;38(10):4588–602. doi: 10.1093/molbev/msab162 (PMC8476148; doi:10.1093/molbev/msab162)
Supplement: msab162_Supplementary_Data [file msab162_supplementary_data.zip › SupportingFigsAndTables.pdf]

Supporting Information

Table S1: Model parameters for unlinked simulation. See Fig. S2 and Methods for parameter definitions.

Model	s	ν_1	ν_2	T	$m_{1\leftarrow 2}$	$m_{2\leftarrow 1}$	θ_S	θ_{NS}
IM	0.93	2.9	2.83	0.084	0.47	0.35	5,992.42	13,842.5

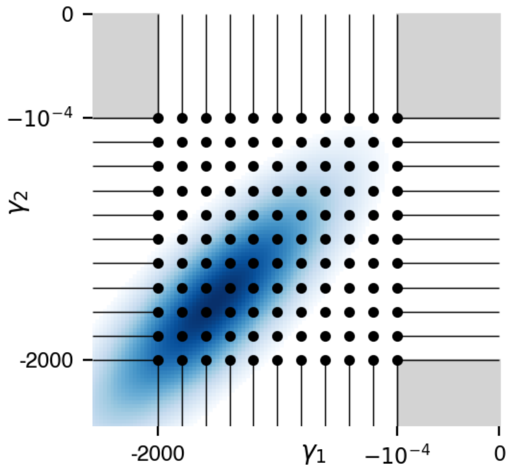

Figure S1: **Illustration of computational approach for calculating expected joint AFS for a given joint DFE.** Dots represent cached frequency spectra. Horizontal and vertical lines indicate single-variable semi-analytic integrations to estimate DFE density outside the sampled domain, and gray regions indicate corresponding double-variable integrations.

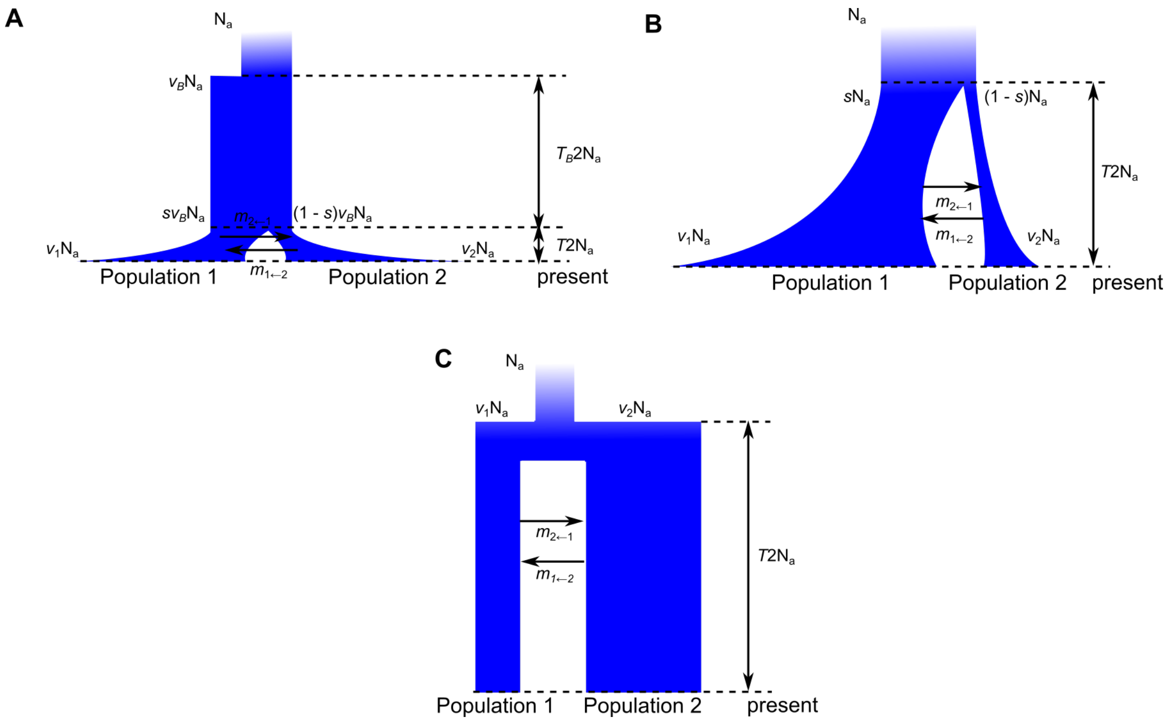

Figure S2: **Demographic models used in this study.** In these models, the population sizes were scaled by the ancestral population size N_a and the times were in units of $2N_a$ generations. The migration rates between Population 1 and 2 can be symmetric ($m_{1\leftarrow 2} = m_{2\leftarrow 1}$) or asymmetric ($m_{1\leftarrow 2} \neq m_{2\leftarrow 1}$). If there was no migration, then $m_{1\leftarrow 2} = m_{2\leftarrow 1} = 0$. **A**: IM_pre model. In this model, the ancestral population experienced an instantaneous population size change at time $T + T_B$ before present. After the change, its population size became $\nu_B N_a$ and remained constant until time T before present. Population 1 and 2 diverged at time T before present and then grew exponentially. For Population 1, its initial population size was $s\nu_B N_a$ and its final population size was $\nu_1 N_a$ at present. For Population 2, its initial population size was $(1-s)\nu_B N_a$ and its final population size was $\nu_2 N_a$ at present. **B**: IM model. This model corresponds to the IM_pre model with no ancestral growth event. **C**: split_mig model. In this model, Population 1 and 2 diverged at time T before present. Their population sizes remained constant after the divergence with $\nu_1 N_a$ for Population 1 and $\nu_2 N_a$ for Population 2.

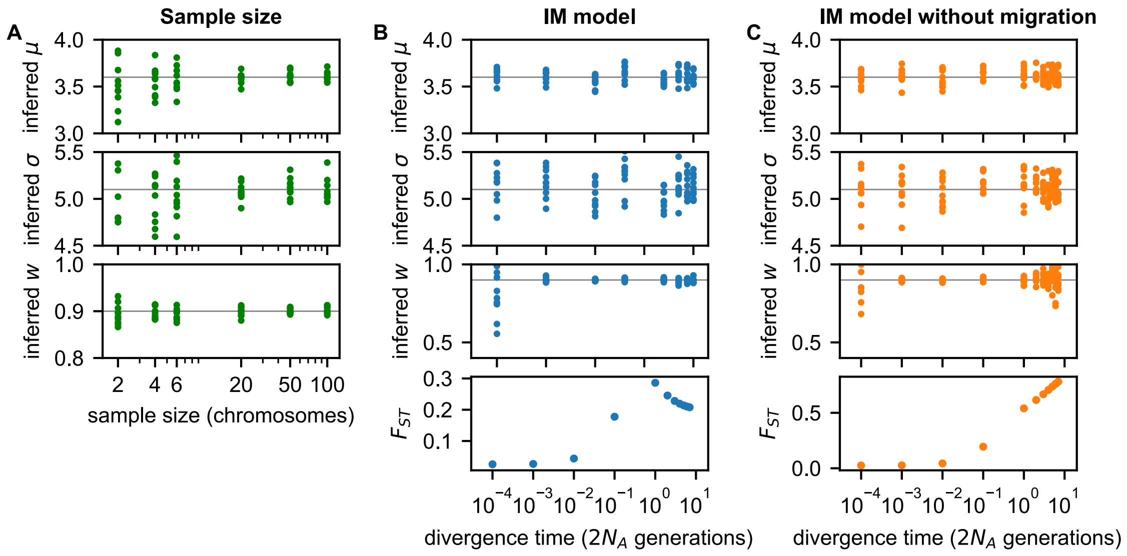

Figure S3: **Precision of joint DFE inference versus sample sizes and divergence time.** Simulated data were generated without linkage and with different sample sizes or divergence times, using the demographic model parameters in Table S1 with logornal $\mu = 3.6$ and $\sigma = 5.1$ and DFE correlation $w = 0.9$. In each panel, points represent inferences from individual data sets and the gray line indicates the true value. For the divergence time analyses, the bottom panels show the divergence statistic F_{ST} expected for synonymous variation under the given demographic scenario (see Table S15 for values). **A**: Inferred DFE parameters for different sample sizes. For small sample sizes, w is still inferred precisely, even though μ and σ are highly uncertain. **B**: Inferred DFE parameters for different divergence times, in a model with migration. Precision of w inference is low for small divergence times. **C**: Inferred DFE parameters for different divergence times, in a model without migration. In this case, precision of w inference is low for both small and large divergence times.

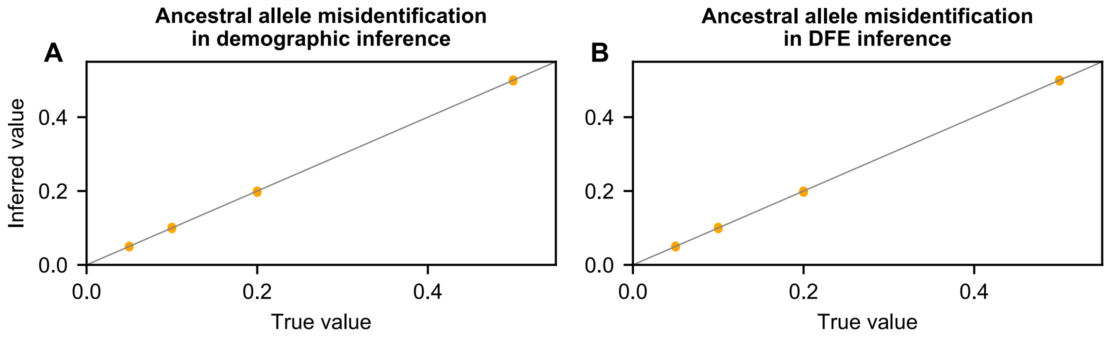

Figure S4: **Accuracy of ancestral allele misidentification in dadi.** To test the accuracy of ancestral allele misidentification in dadi, we simulated data using the demographic model in Table S1 and assumed DFE parameters $\mu = 3.6$, $\sigma = 5.1$, $w = 0.9$. **A**: Accuracy of ancestral allele misidentification in demographic inference. **B**: Accuracy of ancestral allele misidentification in DFE inference. The data plotted in these figures and other inferred demographic or DFE parameters can be found in Table S14.

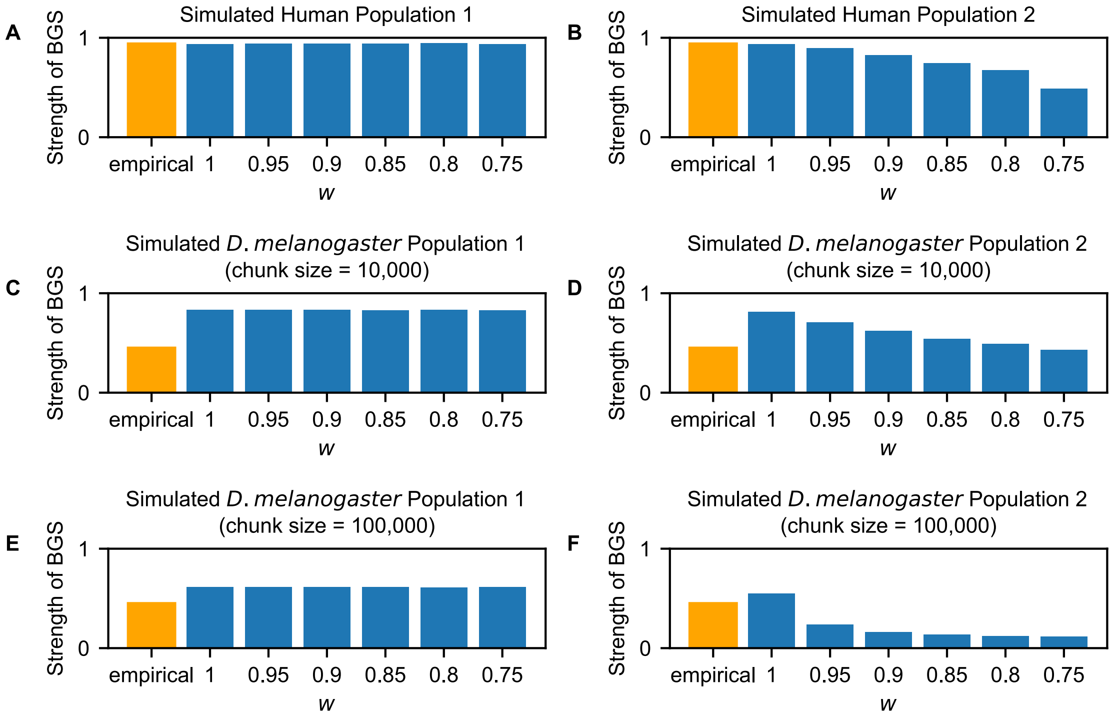

Figure S5: **Strength of background selection (BGS) between simulation and empirical studies.** The strengths of BGS in empirical studies are from Charlesworth (2013). The strengths in simulation are estimated from the observed number of pairwise differences between two chromosomes in the non-neutral scenarios versus the neutral ones. The data plotted in these figures can be found in Table S8.

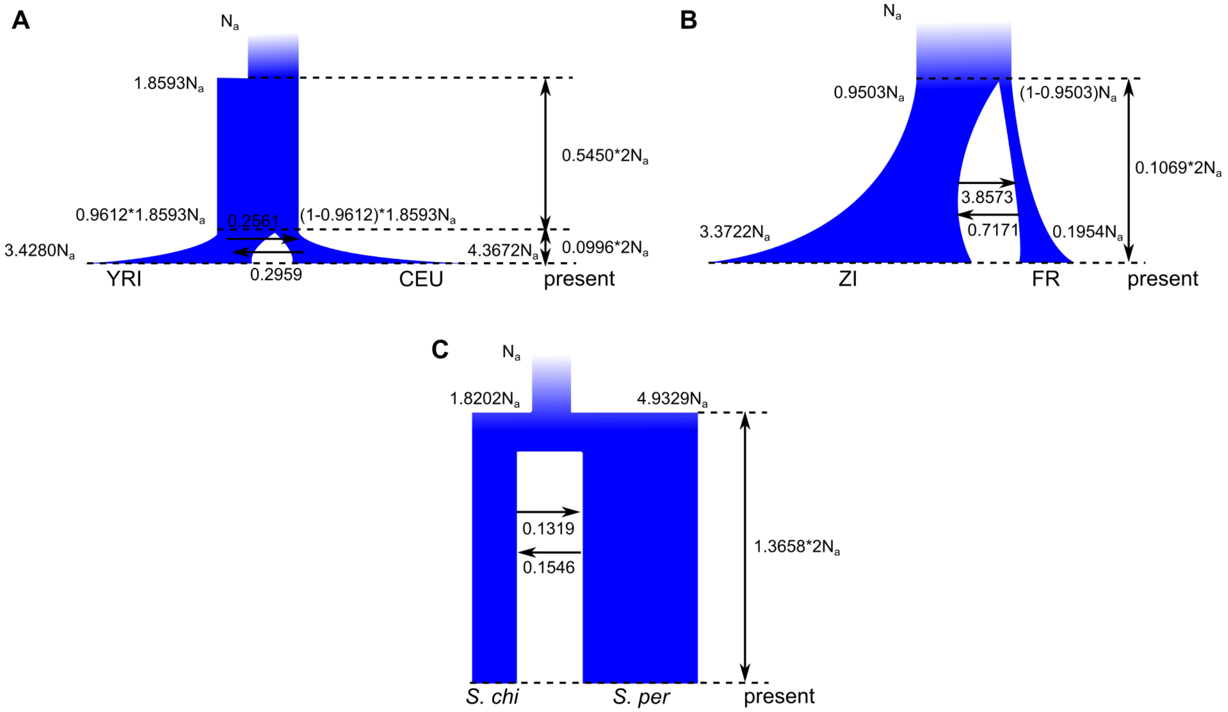

Figure S6: **Best fit demographic models.** **A**: The best fit demographic model for humans. **B**: The best fit demographic model for *D. melanogaster*. **C**: The best fit demographic model for wild tomatoes.

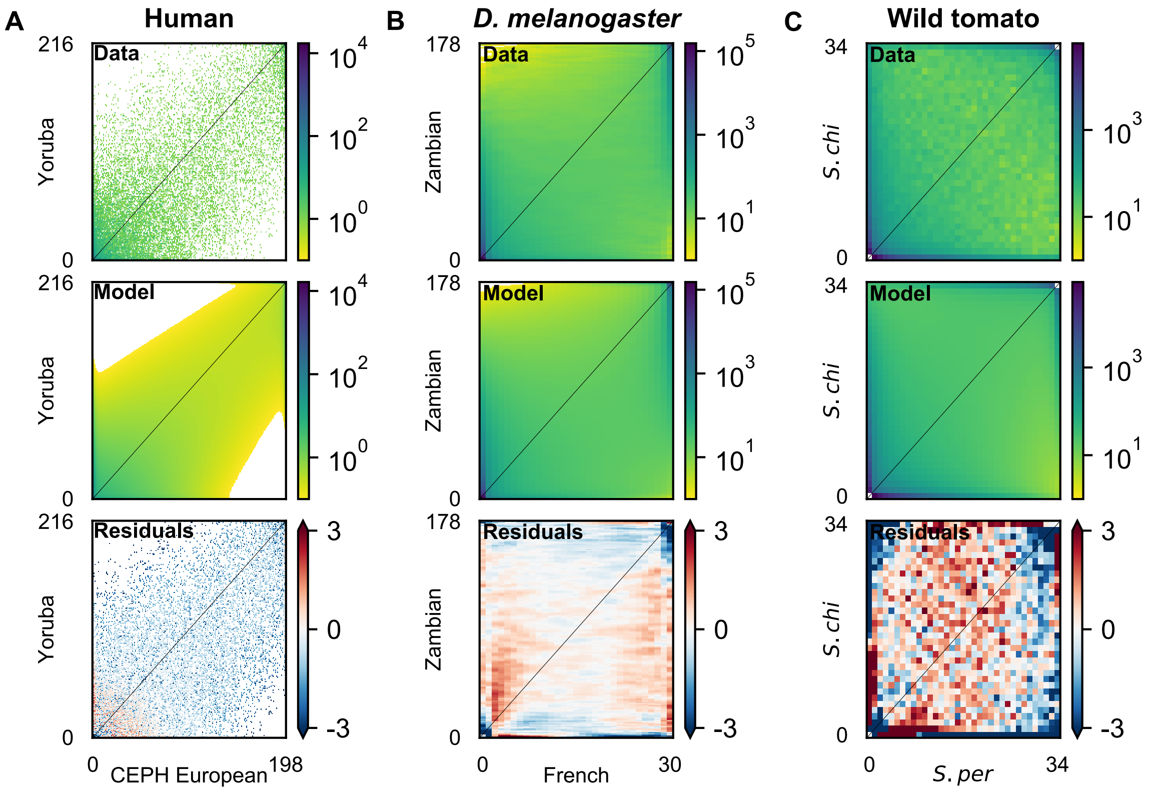

Figure S7: **Model fits to joint allele frequency spectra (AFS) using synonymous data.** **A**: Joint AFS for the human synonymous data and the best fit model (Fig. S6A). **B**: Joint AFS for the *D. melanogaster* synonymous data and the best fit model (Fig. S6B). **C**: Joint AFS for the wild tomato synonymous data and the best fit model (Fig. S6C).

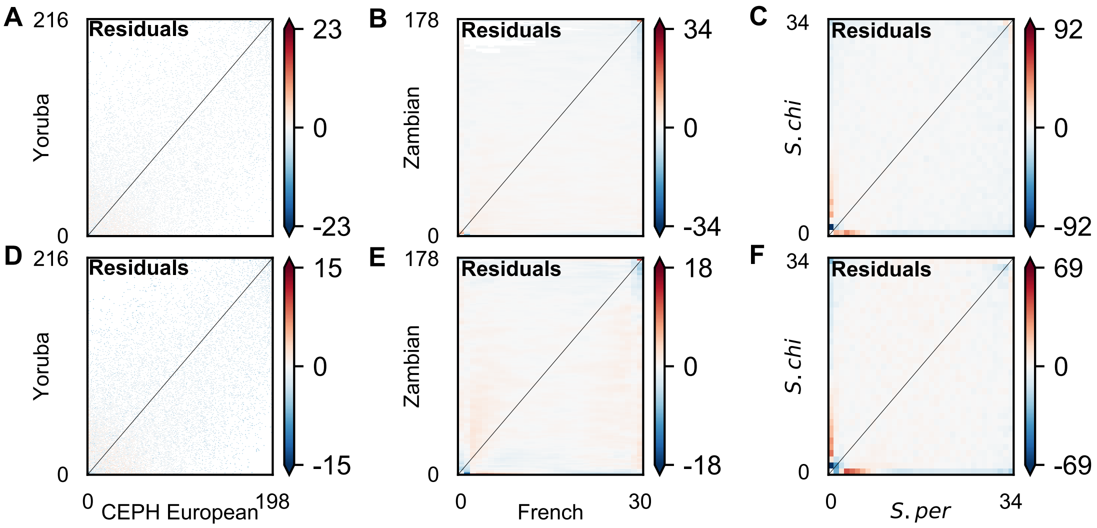

Figure S8: **Residual plots with full range.** **A**: Full-range residual plot of the joint AFS for the human nonsynonymous data and the best fit model. **B**: Full-range residual plot of the joint AFS for the *D. melanogaster* nonsynonymous data and the best fit model. **C**: Full-range residual plot of the joint AFS for the wild tomato nonsynonymous data and the best fit model. **D**: Full-range residual plot of the joint AFS for the human synonymous data and the best fit model. **E**: Full-range residual plot of the joint AFS for the *D. melanogaster* synonymous data and the best fit model. **F**: Full-range residual plot of the joint AFS for the wild tomato synonymous data and the best fit model.

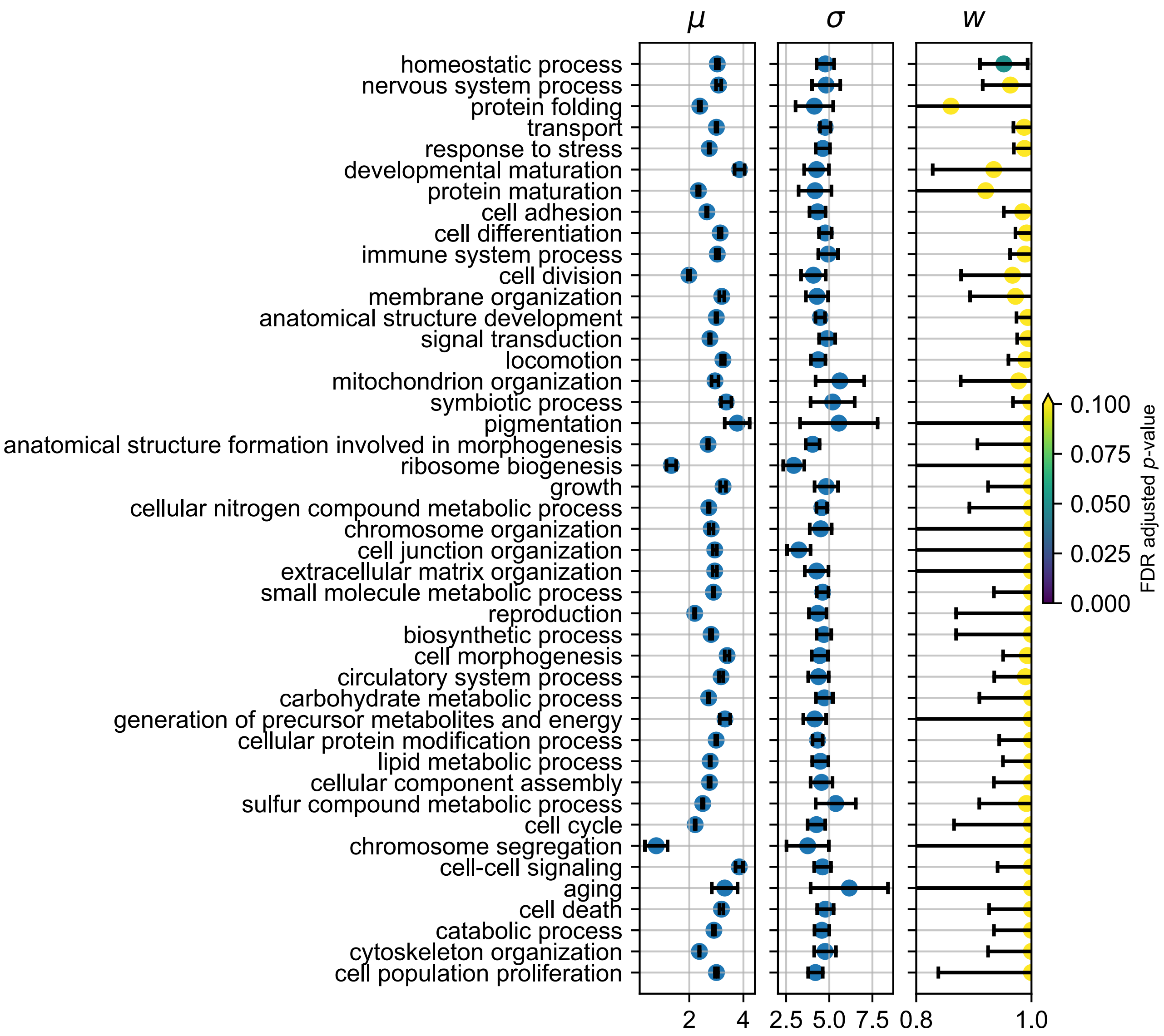

Figure S9: **Joint DFE inference for different GO terms in humans.** Plotted are maximum likelihood inferences with 95% confidence intervals. For inferred w, colors indicate FDR-adjusted p-values from two-tailed z-tests as to whether the confidence interval overlaps $w = 1$. The data plotted in these figures can be found in Table S9.

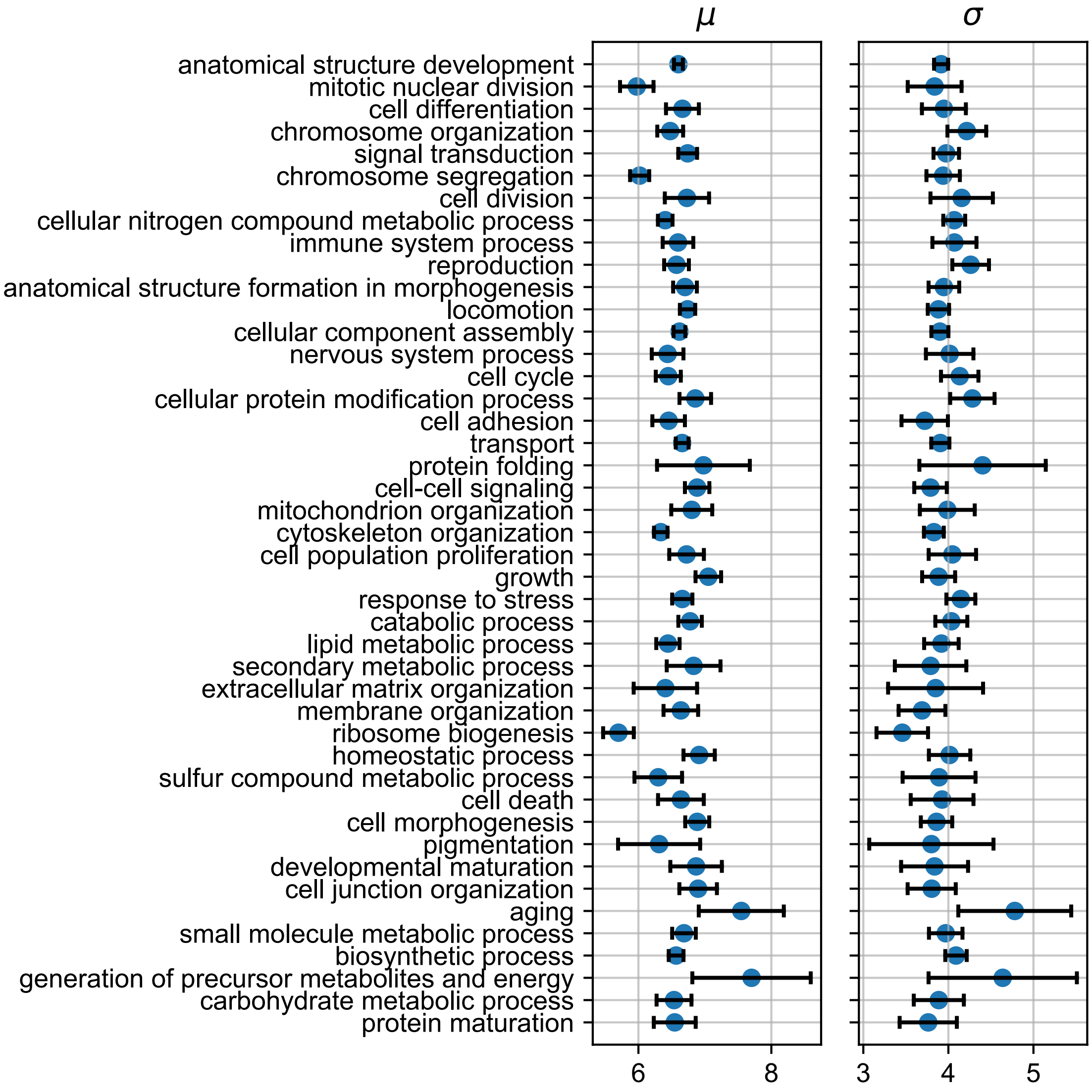

Figure S10: **Inferred μ and σ from the joint DFE inference for different GO terms in *D. melanogaster*.** Plotted are maximum likelihood inferences with 95% confidence intervals. The data plotted in these figures can be found in Table S10.

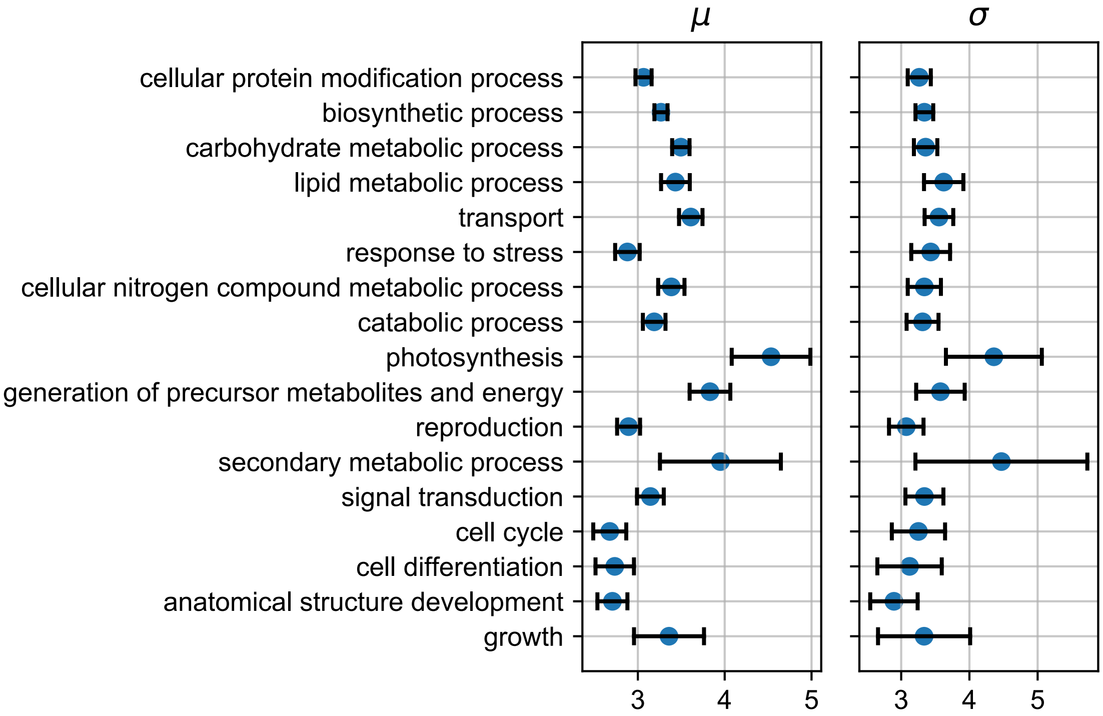

Figure S11: **Inferred μ and σ from the joint DFE inference for different GO terms in wild tomatoes.** Plotted are maximum likelihood inferences with 95% confidence intervals. The data plotted in these figures can be found in Table S11.

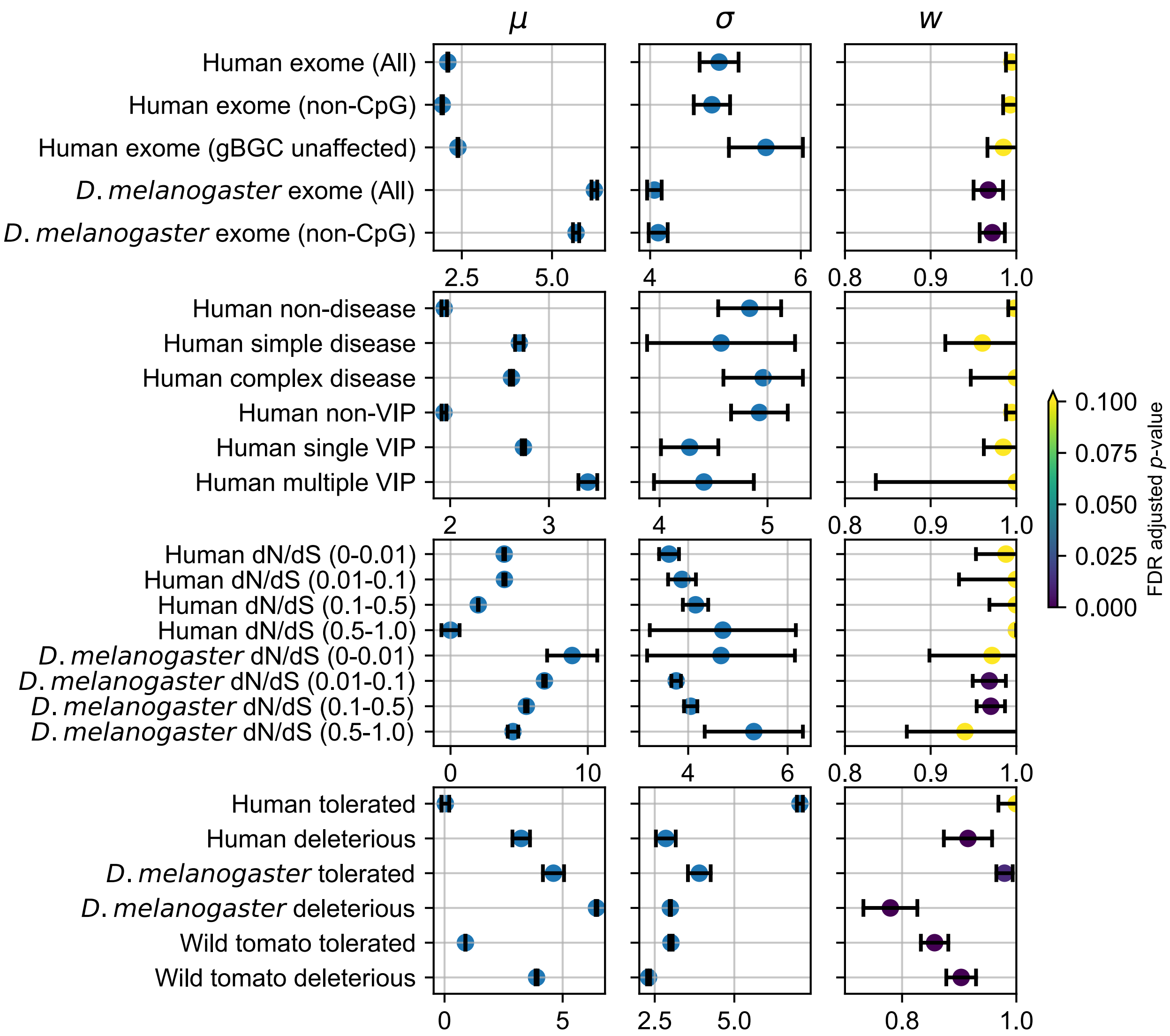

Figure S12: **Joint DFE inference for different types of data in humans and *D. melanogaster*.** Plotted are maximum likelihood inferences with 95% confidence intervals. For inferred w, colors indicate FDR-adjusted p-values from two-tailed z-tests as to whether the confidence interval overlaps $w = 1$. The data plotted in these figures can be found in Table S9, S10 and S11.

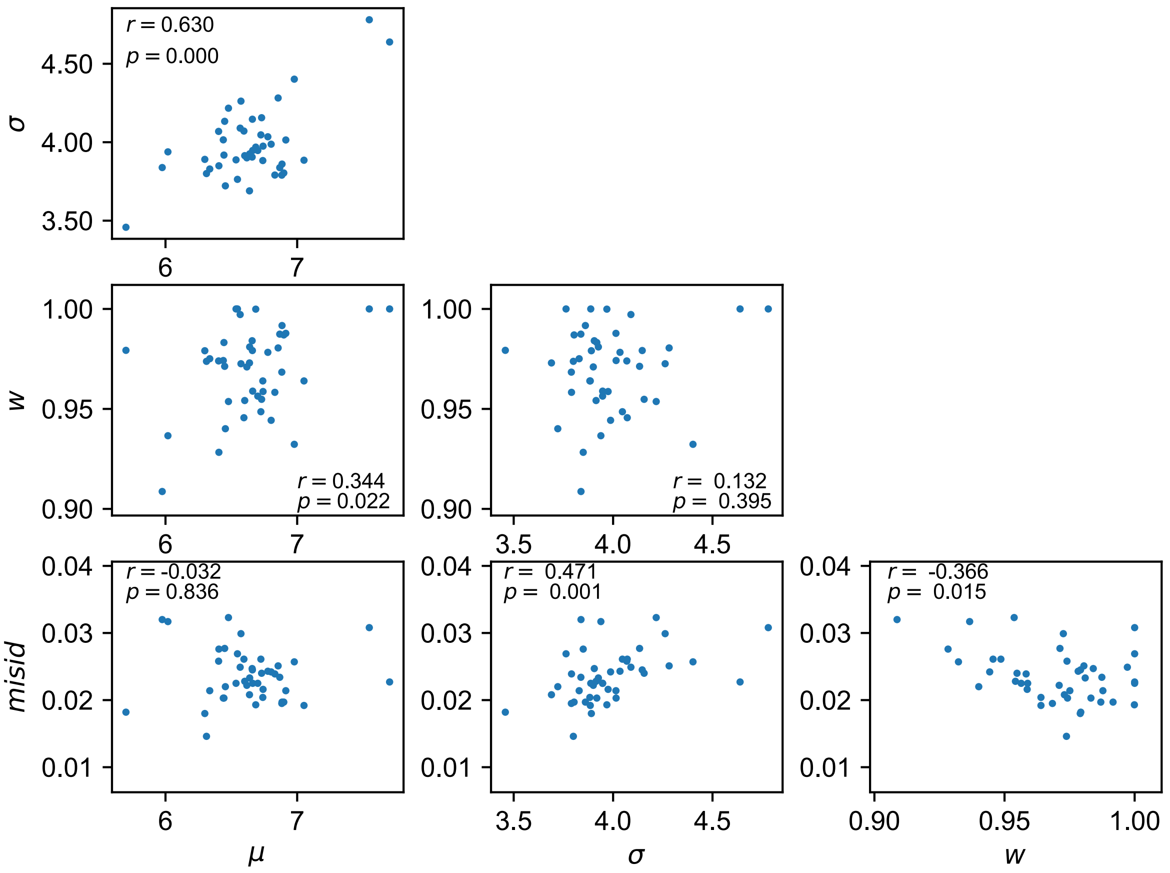

Figure S13: **Relationships among among fitted parameters to *D. melanogaster* GO terms.** Insets indicate Pearson correlations. The data plotted in these figures can be found in Table S10.

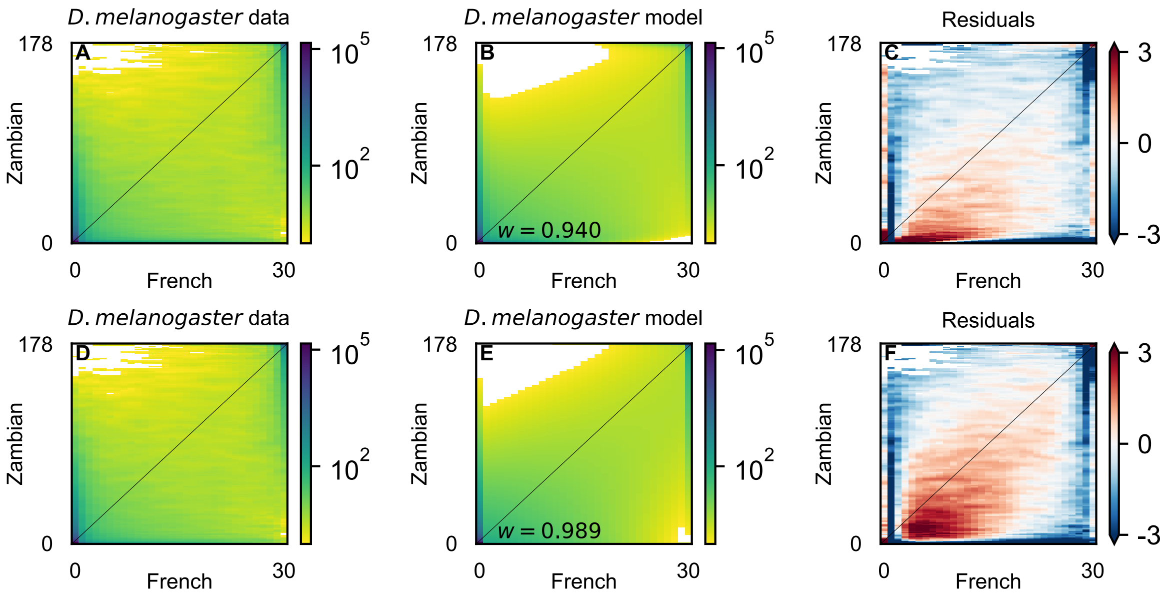

Figure S14: ***D. melanogaster*** **results assuming simpler demographic models.** **A, B & C**: A split_mig model (Fig. S2C) with symmetric migration fits the data less well than our full model with exponential growth and asymmetric migration. (Compare residuals with Fig. S7F.) **D, E & F**: A split_mig model without migration fits even worse.

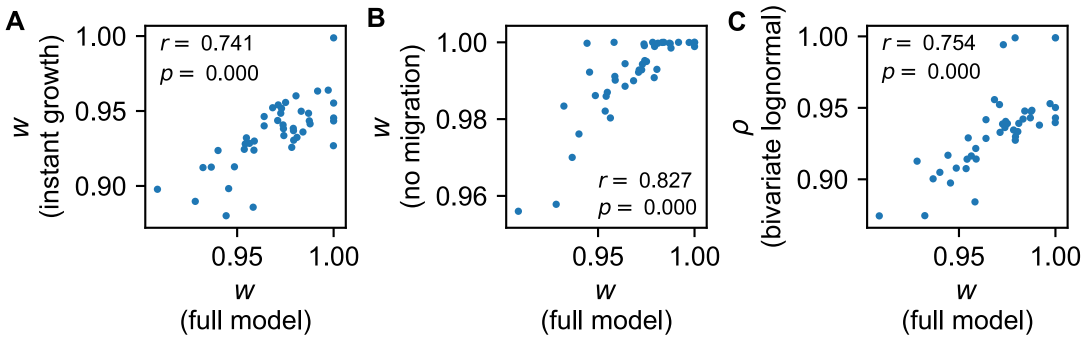

Figure S15: ***D. melanogaster*** **results assuming different models.** For the *D. melanogaster* Gene Ontology terms, inferences from the full demographic model and lognormal mixture DFE model are compared with **A**: A split_mig model with symmetric migration, **B**: A split_mig model withnot migration and **C**: A DFE model with a bivariate lognormal distribution. The data plotted in these figures can be found in Table S12.

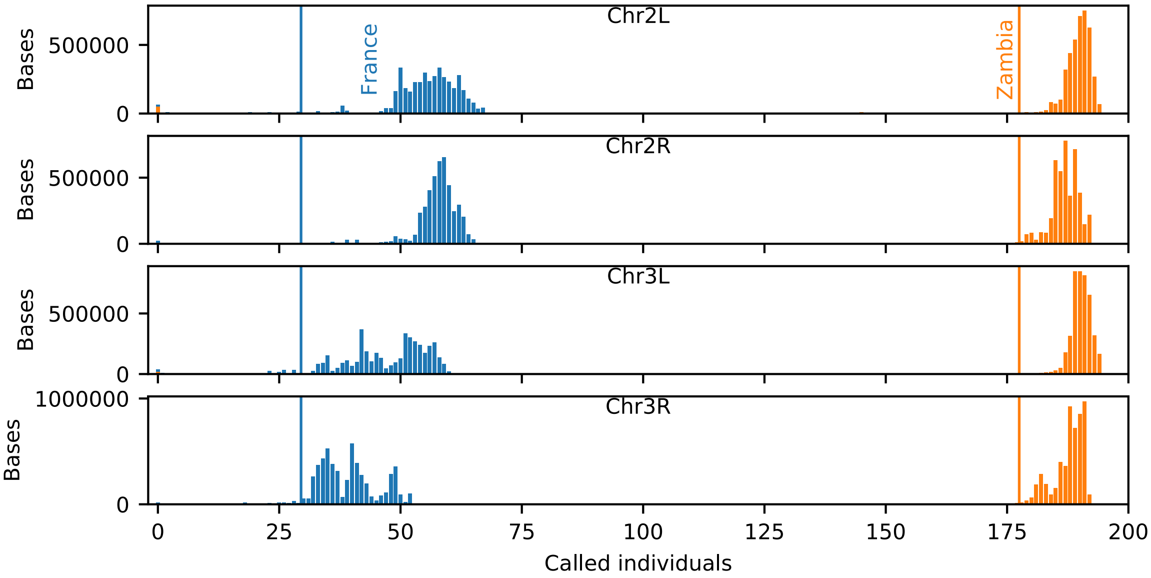

Figure S16: **Calling rate for different chromosome arms in analyzed *D. melanogaster* data.** For each arm, histograms indicate the number of bases at which a given number of individuals were called in each population. The vertical lines indicate the projection sizes for the AFS analysis.

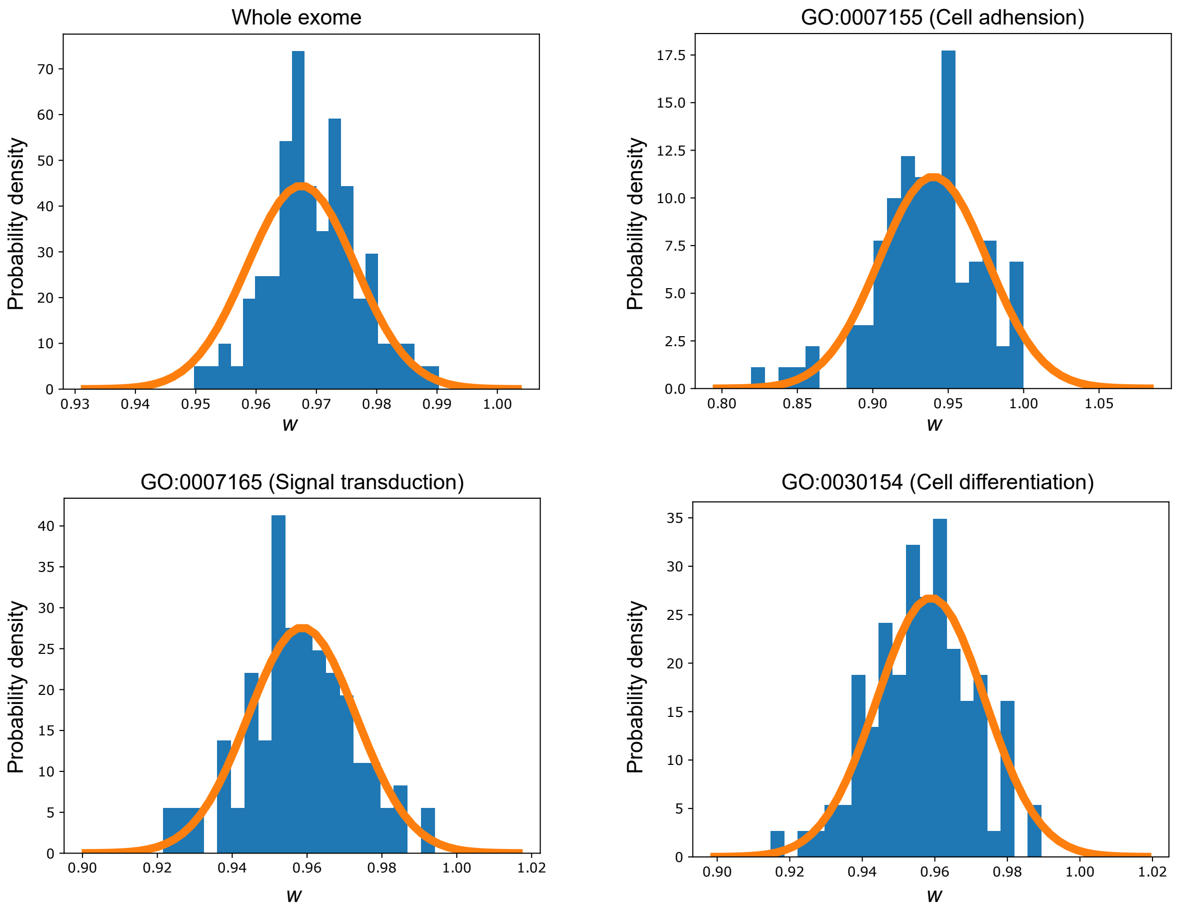

Figure S17: **Comparison between uncertainties estimated with the Godambe Information Matrix and bootstrap fitting.** For the *D. melanogaster* data, each panel shows a different subset of genes/mutations. In each panel, the histogram shows results from conventional bootstrap fitting, while the smooth curve is a normal distribution centered at the maximum likelihood inferred value and standard deviation estimated using the Godambe approach.

Table S2: Full results for sample size precision tests (Fig. S3A).

Table S3: Full results for split time precision tests (Fig. S3B & C).

Table S4: Full results for demographic history robustness tests (Fig. 2A).

Table S5: Full results for dominance robustness tests (Fig. 2B).

Table S6: Full results for DFE distribution robustness tests (Fig. 2C & D).

Table S7: Full results for background selection robustness tests (Fig. 3). Demographic model parameters are defined in Fig. S2.

Table S8: Full results for background selection strength evaluation (Fig. S5). `pi0` and `pi1` denote pairwise diversity in population 1 and 2, respectively. `B0` and `B1` denote the corresponding strengths of background selection, obtained by dividing `pi0` and `pi1` from the simulations with selection by their values in the simulations without selection.

Table S9: Full results for joint DFE inferences in humans (Fig. 4, 5, S9, S12).

Table S10: Full results for joint DFE inferences in *D. melanogaster* (Fig. 4, 5, 6, S10, S12, S13).

Table S11: Full results for joint DFE inferences in wild tomato (Fig. 4, 5, 6, S11, S12).

Table S12: Full results for joint DFE inferences under alternative demographic history and DFE models in *D. melanogaster* (Fig. S14 & S15).

Table S13: DFE inference results with finer integration. Proteome-wide analyses for humans and *D. melanogaster* were repeated with integration performed over 100 values of γ in the range 10^{-4} to 2000, rather than 50 values (Fig. S1). The results are statistically indistinguishable between the two integration approaches.

Table S14: Inferences on simulated data including ancestral state misidentification (Fig. S4).

Table S15: Values of F_{ST} as divergence time is varied in Fig. S3.

Table S16: Comparison of *D. melanogaster* demographic model parameters. Arguello et al. (2019) parameters were taken from the Beijing-Netherlands-Zimbabwe column of their Fig. 3B. G denotes the generation time, which Arguello et al. (2019) assumed to be 0.1 years.

parameter	this work	Arguello et al. (2019)
$(1-s)$	0.050	$0.018 = N_e$ Neth. bottleneck$/N_e$ anc. Africa
ν_{ZI}	3.4	$2.0 = N_e$ Zim. present$/N_e$ anc. Africa
ν_{FR}	0.20	$0.24 = N_e$ Neth. present$/N_e$ anc. Africa
T	0.11	$0.17 = T_{\text{split}}$ Netherlands$/(2N_e$ anc. Africa $G)$
$m_{ZI\leftarrow FR}$	0.72	$2.5 = 2Nm$ ancestral Netherlands to Zimbabwe
$m_{FR\leftarrow ZI}$	3.9	$0.78 = 2Nm$ ancestral Zimbabwe to Netherlands

Table S17: Comparison of human demographic model parameters. Gravel et al. (2011) parameters were taken from the Low-coverage + exons column of their Table 2. G denotes the generation time, which Gravel et al. (2011) assumed to be 25 years.

parameter	this work	Gravel et al. (2011)
ν_B	1.86	$1.98 = N_{AF}/N_A$
T_B	0.55	$0.27 = (T_{AF} - T_B - T_{EU-AS})/(2N_AG)$
$(1-s)\nu_B$	0.04	$0.14 = N_{EU0}/N_A$
ν_{YRI}	3.43	$1.98 = N_{AF}/N_A$
ν_{CEU}	4.37	$4.6 = N_{EU0}/N_A\ (1 + r_{EU}/100)^{(T_{EU-AS}/G)}$
T	0.10	$0.14 = T_B/(2N_AG)$
$m_{YRI\leftarrow CEU}$	0.30	$0.37 = m_{AF-EU}2N_A$
$m_{CEU\leftarrow YRI}$	0.26	$0.37 = m_{AF-EU}2N_A$

Table S18: Comparison of tomato demographic model parameters. Beddows et al. (2017) confidence intervals were taken from their Supplemental Table 4.

parameter	this work	Beddows et al. (2017)
ν_{chi}	1.82	(1.26 - 1.32) = Nu1F
ν_{per}	4.93	(3.69 - 3.87) = Nu2F
T	1.37	(1.46 - 1.56) = Tau
$m_{chi\leftarrow per}$	0.15	(0.13 - 0.14) = m21
$m_{per\leftarrow chi}$	0.13	(0.26 - 0.28) = m12
